# Triazoles synthesis using nanocatalyst triazine–pyrimidine-modified cobalt-based metal–organic frameworks†

**DOI:** 10.1039/d5na00299k

**Published:** 2025-07-21

**Authors:** Mahtab Amirian, Ramin Ghorbani-Vaghei, Sedigheh Alavinia

**Affiliations:** a Department of Organic Chemistry, Faculty of Chemistry and Petroleum Sciences, Bu-Ali Sina University 6517838683 Hamadan Iran rgvaghei@yahoo.com ghorbani@basu.ac.ir; b Department of Organic Chemistry, Faculty of Chemistry, University of Guilan Rasht Iran

## Abstract

This research introduces a recyclable and environmentally friendly catalyst, Co(BDC-NH_2_)–TA–PY, for the efficient synthesis of triazoles *via* the reaction of benzaldehydes, nitromethane, and sodium azide. The synthesis of Co(BDC-NH_2_)–TA–PY was carried out through the post-synthetic modification of Co(BDC-NH_2_), incorporating triazine–pyrimidine (TA–PY) functional groups. The key advantage of this catalyst lies in its dual functionality: the Lewis acidic sites of the Co(BDC-NH_2_) metal–organic framework (MOF) are complemented by the Brønsted basic sites of the triazine and pyrimidine groups. This study represents the first example of a post-synthetic modification of the Co(BDC-NH_2_) metal–organic framework (MOF) by integrating triazine and pyrimidine functional groups, which significantly enhanced its catalytic performance. The abundant TA–PY ligand increased the catalytic activity from 42% to 94%. Co(BDC-NH_2_) nanocrystals were successfully synthesized and characterized, exhibiting a highly crystalline structure. Following the post-synthetic modification to synthesize Co(BDC-NH_2_)–TA–PY, the catalyst retained its uniform morphology, ensuring consistent structural integrity. Furthermore, the structural integrity, morphology, and chemical composition of the reused Co(BDC-NH_2_)–TA–PY were thoroughly examined, revealing no significant differences between the fresh and reused catalysts. These results highlight the potential of functionalized cobalt-based metal–organic frameworks as versatile and sustainable catalysts for broader organic reactions.

## Abstract 2

This research introduces a recyclable and environmentally friendly catalyst, Co(BDC-NH_2_)–TA–PY, for the efficient synthesis of triazoles *via* the reaction of benzaldehydes, nitromethane, and sodium azide.

## Graphical abstract

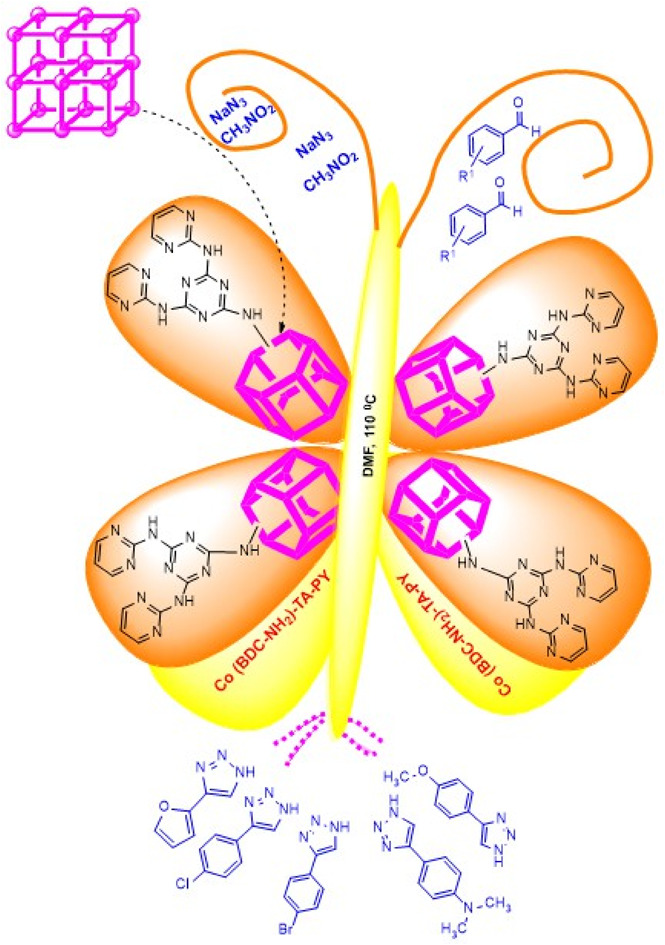

## 1. Introduction

**Scheme 1:**
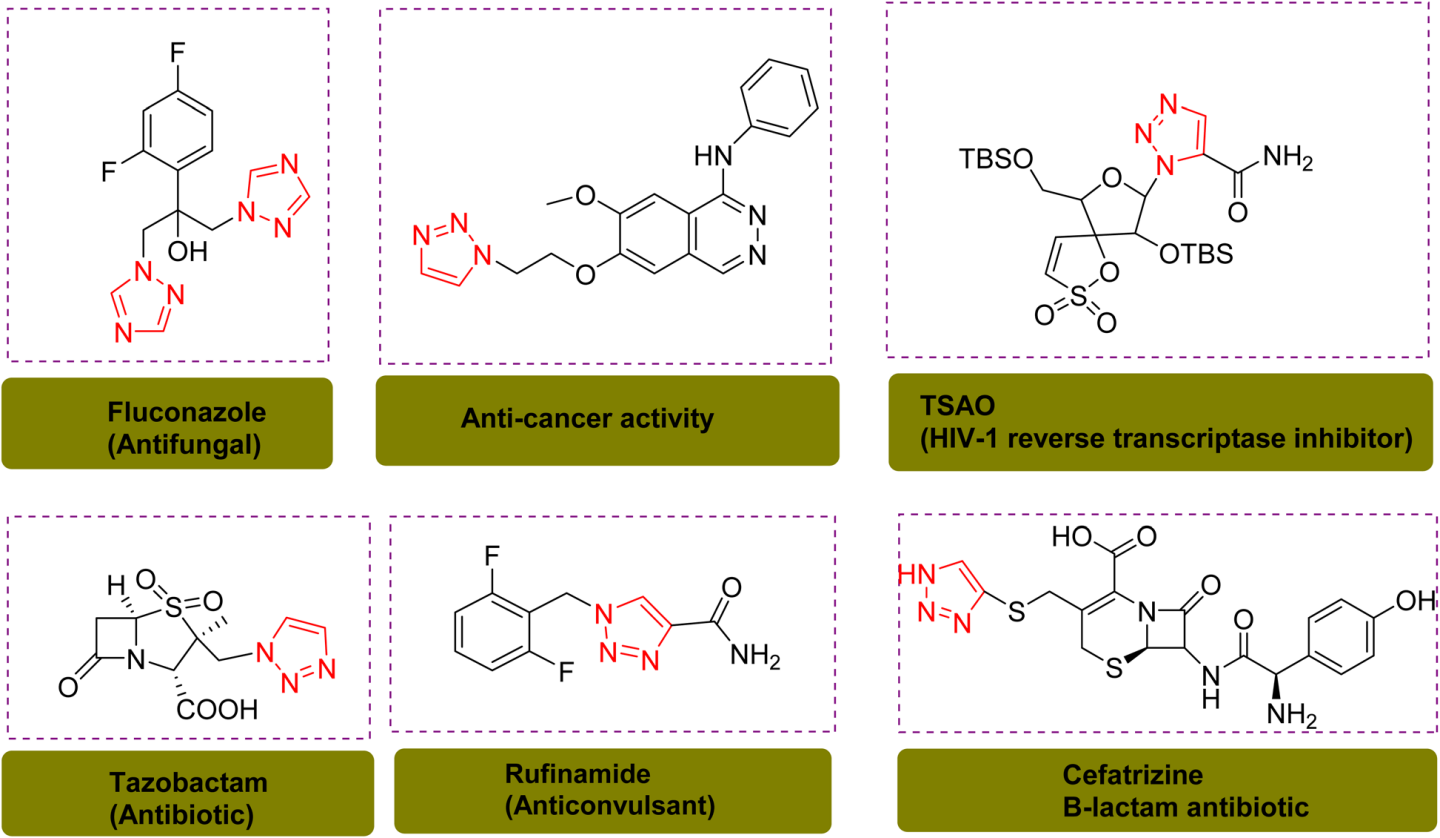
Chemical structure of triazole containing drugs.^9^

Heterocyclic compounds are ubiquitous and versatile organic molecules that play a crucial role in natural and synthetic chemistry. They are integral components of numerous natural products and biologically active compounds, exhibiting a wide range of biological and pharmacological properties. These compounds are essential in the development of pharmaceuticals, agrochemicals, and materials, owing to their diverse structural frameworks and functional versatility. Their presence in key biomolecules and their ability to interact with biological targets make them indispensable in drug discovery and therapeutic applications.^1,2^ Specifically, triazoles and their derivatives are a privileged class of nitrogen heterocyclic scaffolds found in numerous synthetic drugs and natural products.^3,4^ They are widely used because of their excellent anticancer,^4,5^ antitumor^6,7^ antifungal,^8^ anti-inflammatory,^9^ antibacterial,^10^ antituberculosis,^11,12^ and anti-diabetic effects (Scheme 1).^13^

Recognizing such promising features, various synthetic techniques for synthesizing these heterocycles with various substituents have been developed over the past few decades to satisfy the growing therapeutic needs of modern society.^14^ Recent advancements in the Henry reaction have significantly contributed to the field of organic chemistry. A key intermediate in this reaction is α,β-nitrostyrene, which acts as a Michael acceptor and participates in conjugate addition reactions.^15^ These intermediates are versatile synthons, widely employed in the synthesis of various essential compounds, including substituted E-alkenes,^16^ vinyl sulfones,^17^ pyridines,^18^ pyrroles,^19^ and furans.^20^ Additionally, α,β-nitrostyrenes are utilized in Henry reaction-cycloaddition sequences to synthesize 4-aryl-NH-1,2,3-triazoles, further expanding their synthetic utility.^21^ Traditional methods for synthesizing 1,2,3-triazoles often face significant challenges, including the use of hazardous reagents, harsh reaction conditions, and excessive energy consumption. Therefore, the development of an eco-friendly, cost-effective, and heterogeneous catalyst provides advantages in terms of reuse and new possibilities for this kind of reaction.

Metal–organic frameworks (MOFs) are an innovative class of porous crystalline polymers.^22–27^ They possess metallic and organic sections, structured in three dimensions. Their distinctive characteristics, such as large pore size, high porosity, high surface area, and proper thermal, chemical, and mechanical stability, make them suitable for many different uses.^28–32^ Through post-synthetic modification and tailored synthesis, MOF-based nanocomposites have been engineered into advanced hybrid catalytic materials, showcasing enhanced activity and recyclability. These developments highlight the significant potential of MOFs in driving efficient and sustainable organic transformations.^33^ The current trend in organic synthesis is to use MOF-based catalysts for environmentally friendly reactions that yield high-purity products. As a result, the development of highly efficient and stable catalysts is crucial for the practical synthesis of triazole-based heterocyclic compounds.

Drawing inspiration from the insights, our objective in this study is to utilize the distinctive characteristics of Co(BDC-NH_2_) MOF structures to prepare Co(BDC-NH_2_)–TA–PY nanocatalyst for efficiently synthesizing triazole compounds. Specifically, we introduced a new functionalization process for the modification of Co(BDC-NH_2_) MOF using triazine and pyrimidine moieties. Our focus lies on producing green MOFs with enhanced catalytic properties for organic reactions. Moreover, its ability to synthesize new triazole derivatives under mild reaction conditions suggests that Co(BDC-NH_2_)–TA–PY can effectively accelerate the organic process.

## 2. Experimental section

### 2.1. Materials and method

Chemicals were purchased from Sigma-Aldrich and Merck in high purity. All the materials (2-aminoterephthalic acid, cobalt(ii) nitrate, DMF, ethanol, 2-aminopyrimidine, cyanuric chloride, acetonitrile, benzaldehyde derivatives, sodium azide, nitromethane, ethyl acetate, *n*-hexane) were of commercial reagent grade and were used without further purification.

### 2.2. Procedure for assembling triazine-pyrimidine (TA–PY) into Co(BDC-NH_2_) (Co(BDC-NH_2_)–TA–PY)

**Scheme 2:**
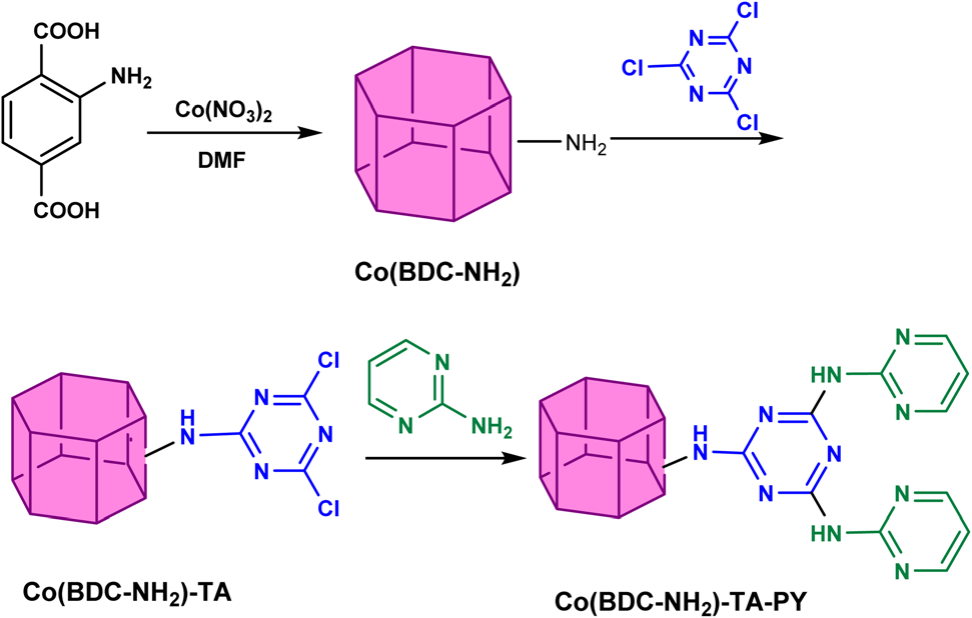
The synthesis procedure of Co(BDC-NH_2_)–TA–PY.

Co(BDC-NH_2_) was synthesized according to the literature in solvothermal conditions.^34^ The synthesis of the Co(BDC-NH_2_)–TA–PY was achieved by the reaction of Co(BDC-NH_2_) (1 g) with cyanuric chloride (1.5 g) using acetonitrile (20 mL) and stirred for 24 h. Afterward, pyrimidine (1.5 g) was added slowly. The final synthesis solution was left to stir at room temperature for 24 h. Afterwards, the reaction mixture was centrifuged at 30 000 rpm for 15 min, and the collected Co(BDC-NH_2_)–TA–PY nanoparticles were washed four times with DMF (for 15 min each). Finally, an ethanol wash was performed for solvent exchange, and the nanocrystals were dried overnight at 40 °C (Scheme 2).

### 2.3. Catalyst characterization

All melting points were determined in a capillary tube on a Boetius melting point microscope. Infrared (IR) spectroscopy was conducted on a PerkinElmer GX FT-IR spectrometer. The thermal properties of the samples were conducted using a thermogravimetric analyzer (TGADSC/Mettler Toledo) ranging from room temperature to 600 °C at a rate of 10 °C min^−1^ under N_2_ atmosphere. The approximate sample weight was 10 mg in TG experiment with 10 °C min^−1^ heating rate. Moreover, ^1^H NMR and ^13^C NMR spectra were obtained on Bruker 400 MHZ spectrometer with DMSO-d_6_ as solvent using tetramethylsilane (TMS) as an internal standard. X-ray diffraction (XRD) data were collected using a Rigaku model Ultima-IV diffractometer and Cu-Kα radiation (1.5405 Å) at 40 kV and 25 mA over a 2*θ* range of 10° to 90°. Scanning electron microscopy (SEM) samples were created by pouring ethanolic solutions onto alumina stubs and coating them with gold using an automatic gold coater (Quorum, Q150T E). The energy-dispersive X-ray spectra (EDS) used for the elemental analysis and mapping were obtained using an Oxford Instruments INCA attachment on a Lyra 3 SEM (dual beam) from TESCAN. TEM images were obtained using a JEM2100F transmission electron microscope from JEOL. The TEM samples were prepared by dropping them from an ethanolic suspension onto a copper grid and allowing them to dry at room temperature. Inductively coupled plasma optical emission spectrometry was used to determine the amount of cobalt particles in the catalyst (ICP-OES; PlasmaQuant PO 9000 – Analytik Jena). The catalyst samples were initially digested in a dilute mixture of HNO_3_ and HCl. Ir calibration curves were produced using standard solutions (ICP Element Standard Solutions, Merck). All adsorption and desorption measurements were performed on a Micromeritics TriStar 3020 version 3.02 (N_2_) system and measured at 77 K. The data were analyzed using the TriStar II 3020 V1.03 software (Micromeritics, Norcross, GA). Prior to the surface area analysis the samples were activated in high vacuum at 80 °C for 12 h. It should be noted that Co(BDC-NH_2_) was treated at 120 °C under high vacuum for 12 h. The pore size distributions were calculated from the adsorption–desorption isotherms.

### 2.4. Catalytic procedure for the preparation of 4-aryl-NH-1,2,3-triazoles in the presence of Co(BDC-NH_2_)–TA–PY

**Scheme 3:**
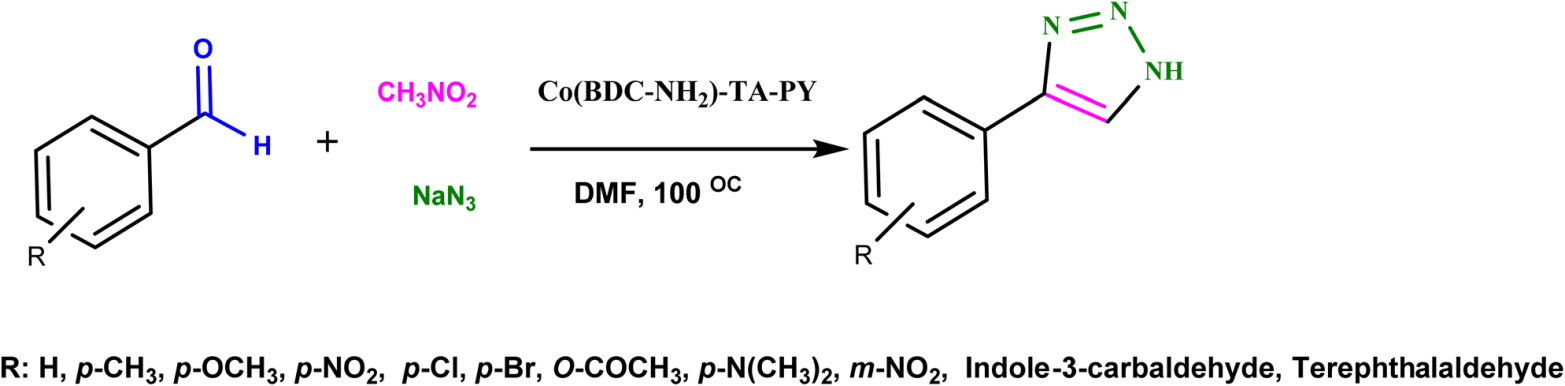
Schematic application of Co(BDC-NH_2_)–TA–PY nanocatalyst for synthesizing 4-aryl-NH-1,2,3-triazoles.

A mixture of benzaldehyde derivatives (1.0 mmol), nitromethane (1.0 mmol), sodium azide (1.0 mmol), and Co(BDC-NH_2_)–TA–PY (30 mg) was heated in DMF (2 mL) at 100 °C. Thin-layer chromatography (TLC) (10 : 3, *n*-hexane and ethyl acetate) was employed to monitor the progress of the reaction. Subsequently, the solution was cooled to room temperature. The product was obtained through gradual evaporation at room temperature and then washed with a mixture of diethyl ether and water (1 : 1, 20 mL) after centrifugation of the Co(BDC-NH_2_)–TA–PY nanocomposite (Scheme 3).

## 3. Results and discussion

### 3.1. Characterization of Co(BDC-NH_2_)–TA–PY nanocatalyst

#### 3.1.1. FT-IR analysis

**Fig. 1:**
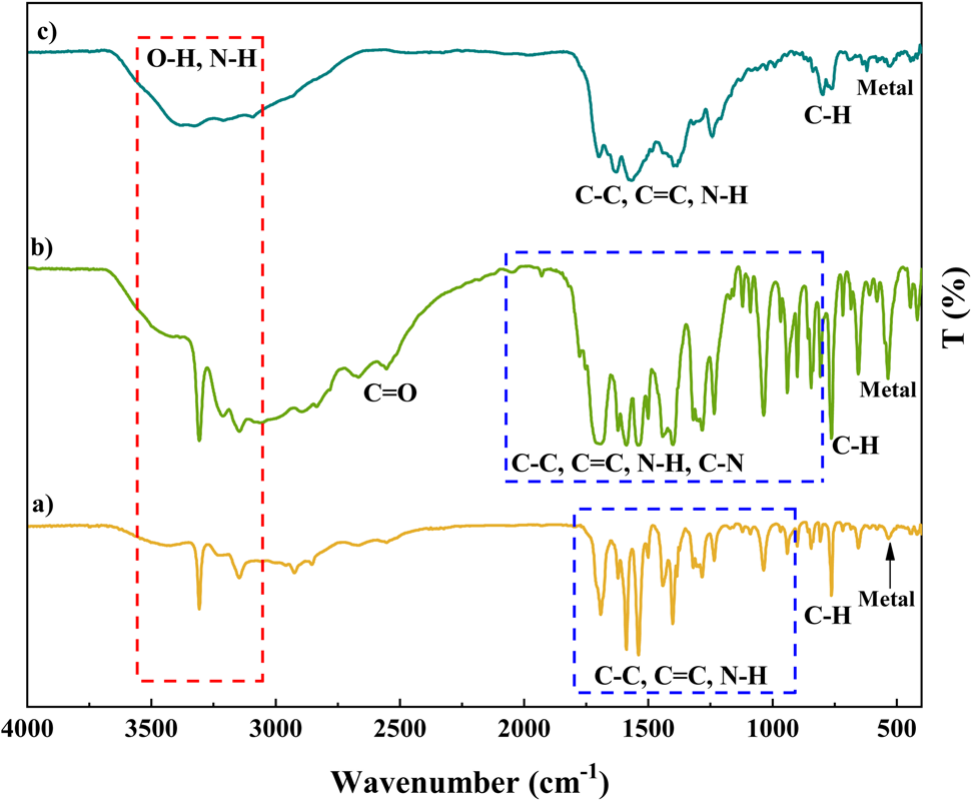
FT-IR analysis of (a) Co(BDC-NH_2_), (b) Co(BDC-NH_2_)–TA, and (c) Co(BDC-NH_2_)–TA–PY.

FT-IR spectra of cobalt-based metal–organic frameworks, Co(BDC-NH_2_)–TA, and as-prepared Co(BDC-NH_2_)–TA–PY revealed several characteristic bands (Fig. 1). In spectrum (a), it is related to Co(BDC-NH_2_), in which the peaks at wavenumbers 530 cm^−1^ and 764 cm^−1^ indicate the presence of metal and C–H bands. Also, in the wavenumber range between 936 cm^−1^ and 1684 cm^−1^, the N–H, C=C, and C–C bands present in the MOF ligand are seen. The peak at wavenumber 3307 cm^−1^ is also related to the O–H band. In spectrum (b), it is related to Co(BDC-NH_2_)–TA, and the presence of peaks at wavenumbers 539 cm^−1^ and 763 cm^−1^ indicates the presence of metal and C–H bands. Additionally, in the wavenumber range between 936 cm^−1^ and 1684 cm^−1^, the C–N, N–H, C=C, and C–C bands are seen. The peak at wavenumber 2546 cm^−1^ is related to the C=O band, and the peak at 3311 cm^−1^ is related to the O–H band. In spectrum (c), the presence of peaks at wavenumbers 526 cm^−1^ and 810 cm^−1^ indicates the presence of metal and C–H bands. In addition, the peaks at wavenumbers 1244 cm^−1^, 1401 cm^−1^, and 1580 cm^−1^ indicate N–H, C=C, and C–C bands, respectively. Also, the presence of a peak at wavenumber 3403 cm^−1^ indicates the O–H and N–H bands.

#### 3.1.2. XRD analysis

**Fig. 2:**
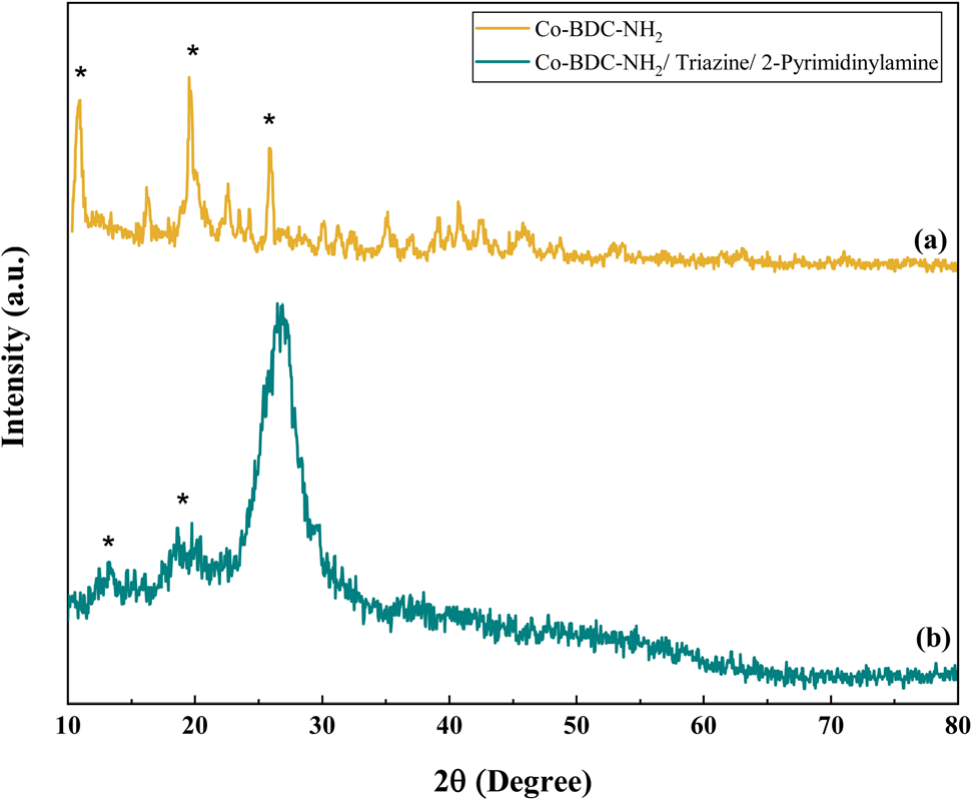
XRD analysis of (a) Co(BDC-NH_2_), and (b) Co(BDC-NH_2_)–TA–PY.

The crystallographic phase information of the synthesized cobalt-based MOFs, and Co(BDC-NH_2_)–TA–PY catalyst was characterized through PXRD analysis (Fig. 2). The XRD pattern of Co(BDC-NH_2_) confirms the successful synthesis of the Co(BDC-NH_2_) metal–organic framework (MOF) and validates its crystalline structure, which aligns with previously reported patterns. The consistency in the PXRD data indicates that the synthesized material retains the expected structural integrity and phase purity.^35^ The prominent diffraction peaks at 2*θ* values of 10.79°, 19.54°, and 25.94° confirm the successful synthesis of crystalline Co(BDC-NH_2_) phases (Fig. 2a). The broad characteristic diffraction peaks in the 2*θ* range of 25–29° (ref. 36) can be assigned to the diffraction characteristic peaks associated with triazine functional groups. The introduction of TA–PY moieties leads to a decrease in the peak intensities associated with the prominent planes of the Co(BDC-NH_2_) MOF.

#### 3.1.3. FE-SEM analysis

**Fig. 3:**
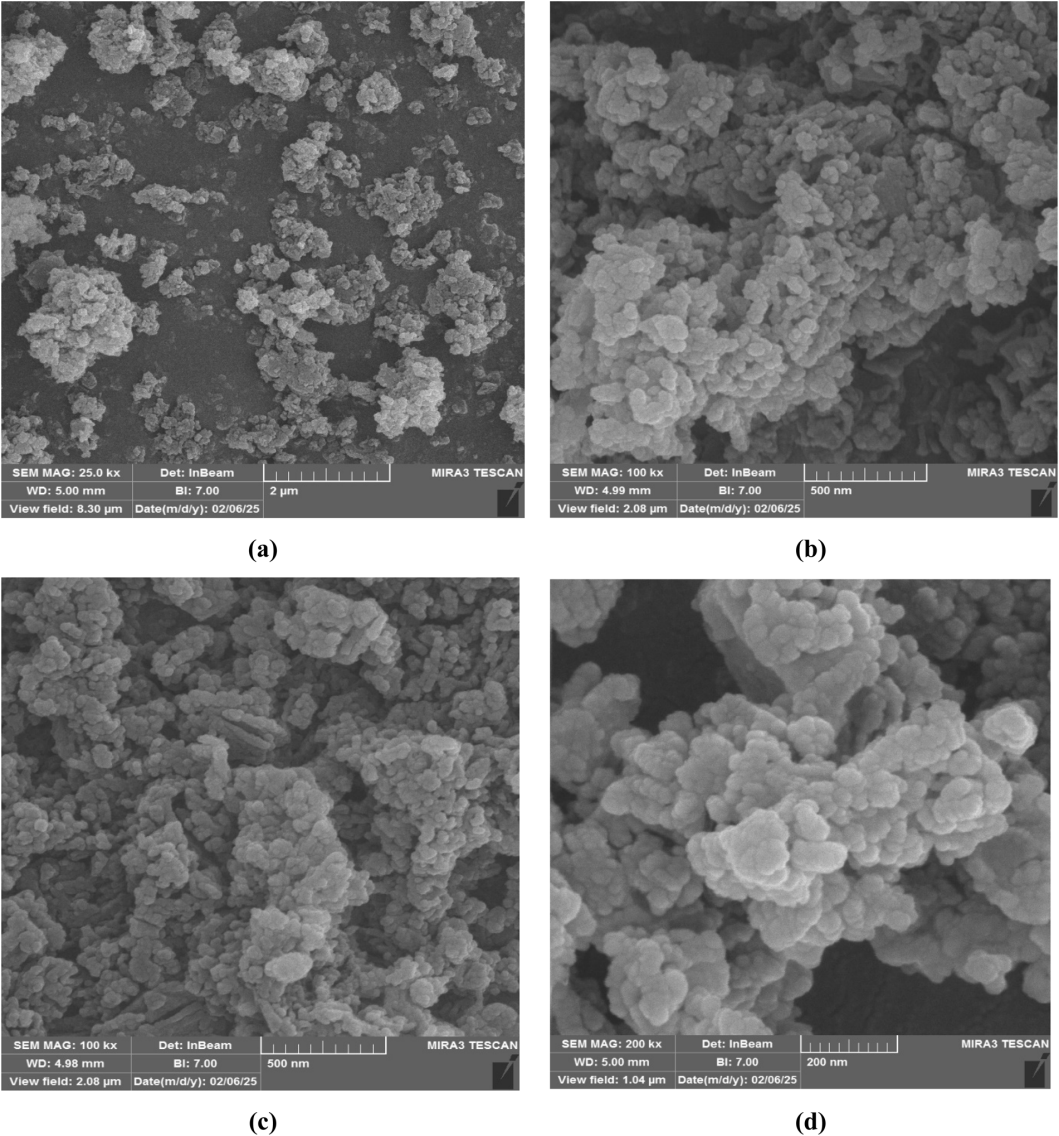
FE-SEM images of Co(BDC-NH_2_)–TA–PY in 2μm (a), 500 nm (b and c), and 200 nm (d).

The morphology and size of the Co(BDC-NH_2_)–TA–PY nanoparticles were investigated using FESEM analysis (Fig. 3). The images indicate that the Co(BDC-NH_2_)–TA–PY nanocatalyst has regular nanoparticles with a rough and porous morphology. This characteristic suggests a substantial surface area, which is crucial for enhancing catalytic applications.

#### 3.1.4. TEM analysis

**Fig. 4:**
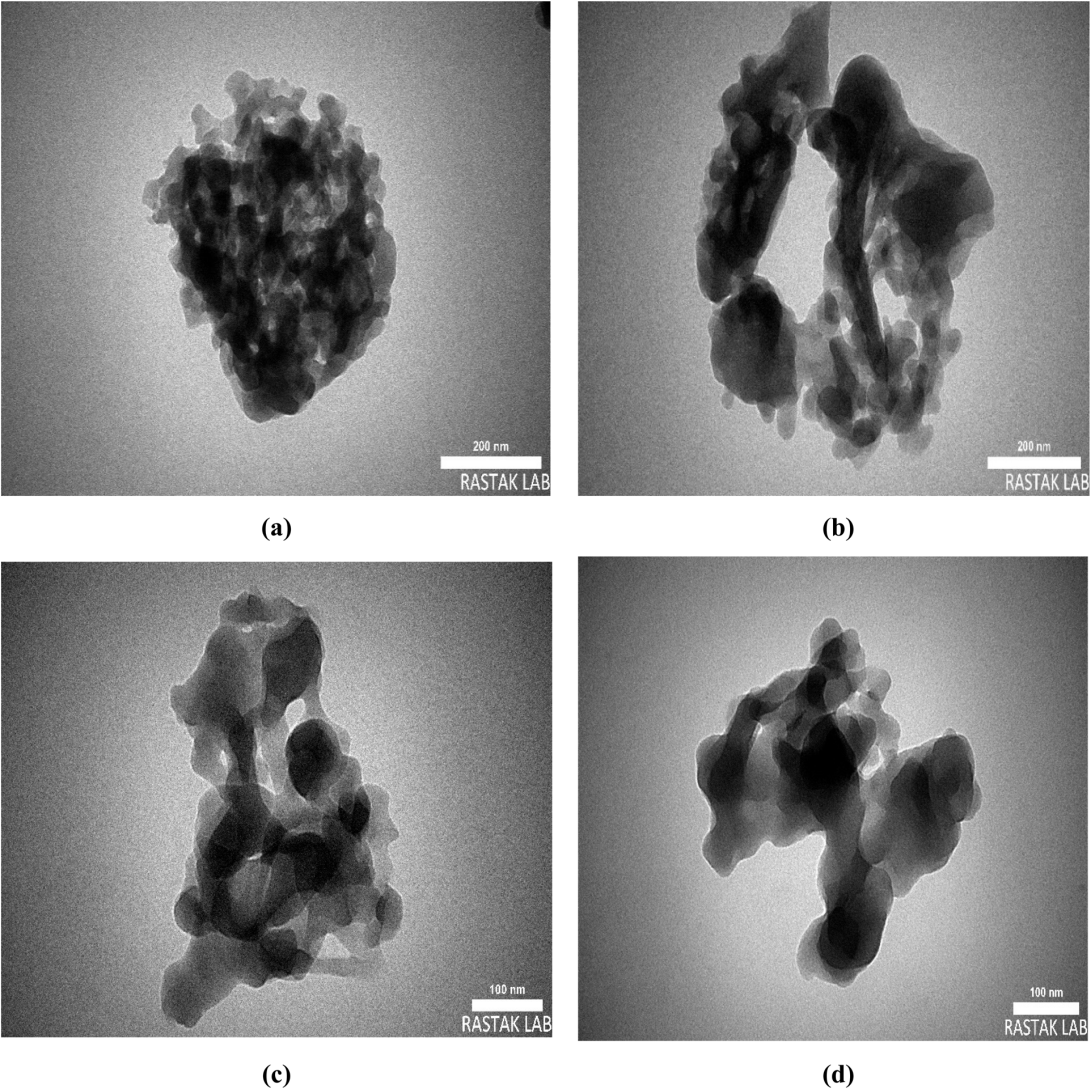
TEM images of Co(BDC-NH_2_)–TA–PY in 200 nm (a and b) and 100 nm (c and d).

TEM images of Co(BDC-NH_2_)–TA–PY demonstrated coexisting spherical nanoparticles and uniform porous structures, indicating a successful post-synthetic modification process. As can be seen from the TEM images, the proper morphology indicates Co(BDC-NH_2_)–TA–PY would be an efficient catalyst for carrying out organic reactions (Fig. 4).

#### 3.1.5. EDS analysis

**Fig. 5:**
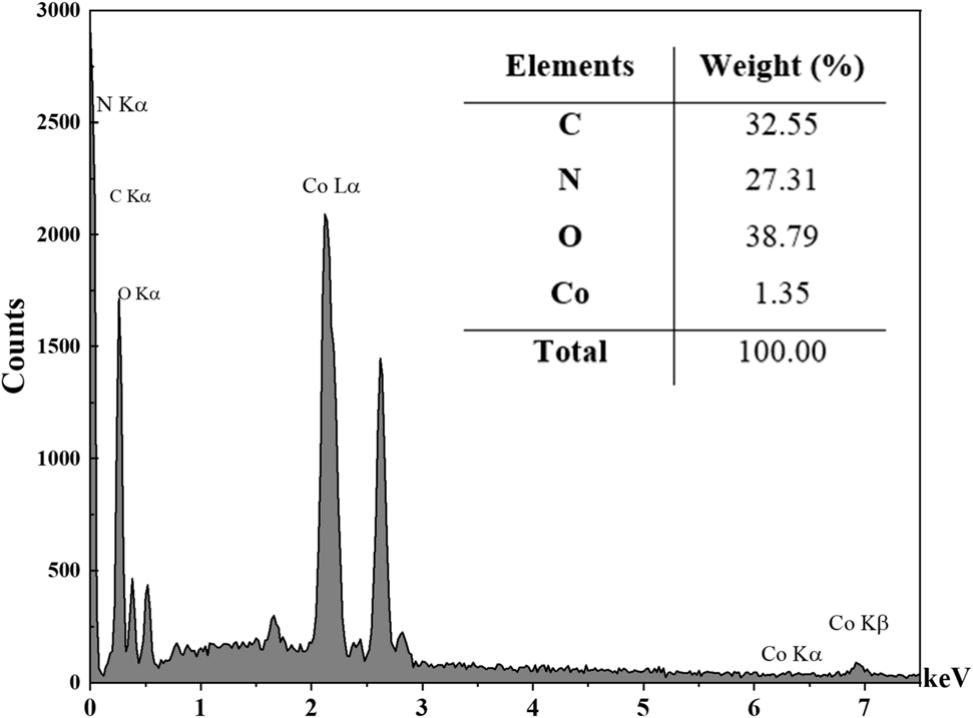
EDX patterns of Co(BDC-NH_2_)–TA–PY.

The EDS results indicated that the Co(BDC-NH_2_)–TA–PY nanocatalyst comprised cobalt, carbon, and nitrogen. The EDS spectrum of the Co(BDC-NH_2_)–TA–PY sample revealed that carbon is the main constituent of the synthesized nanocatalyst. Nitrogen is present in the amino groups of BDC-NH_2_, the triazine (TA) moiety, and the pyrimidine (PY) group, confirming the incorporation of these functional groups. Carbon is derived from the organic framework, TA, and PY functional groups. Cobalt is associated with the metal of the Co(BDC-NH_2_)–TA–PY nanocatalyst. Lastly, oxygen is typically present in the carboxylate groups of BDC-NH_2_ and possibly in any residual solvents or adsorbed water molecules (Fig. 5 and Table 1).

**Table 1:** The quantitative information of EDS spectrum of Co(BDC-NH_2_)–TA–PY nanocatalyst

Elements	Int	Errors	*K*	*K*_r_	Wt%	At%
C	86.7	41.8326	0.6161	0.1642	32.55	38.13
N	21.7	41.8326	0.1553	0.0414	27.31	27.43
O	26.3	41.8326	0.1900	0.0506	38.79	34.11
Co	3.8	0.3513	0.0387	0.0103	1.35	0.32
Total			1.0000	0.2665	100.00	100.00

**Fig. 6:**
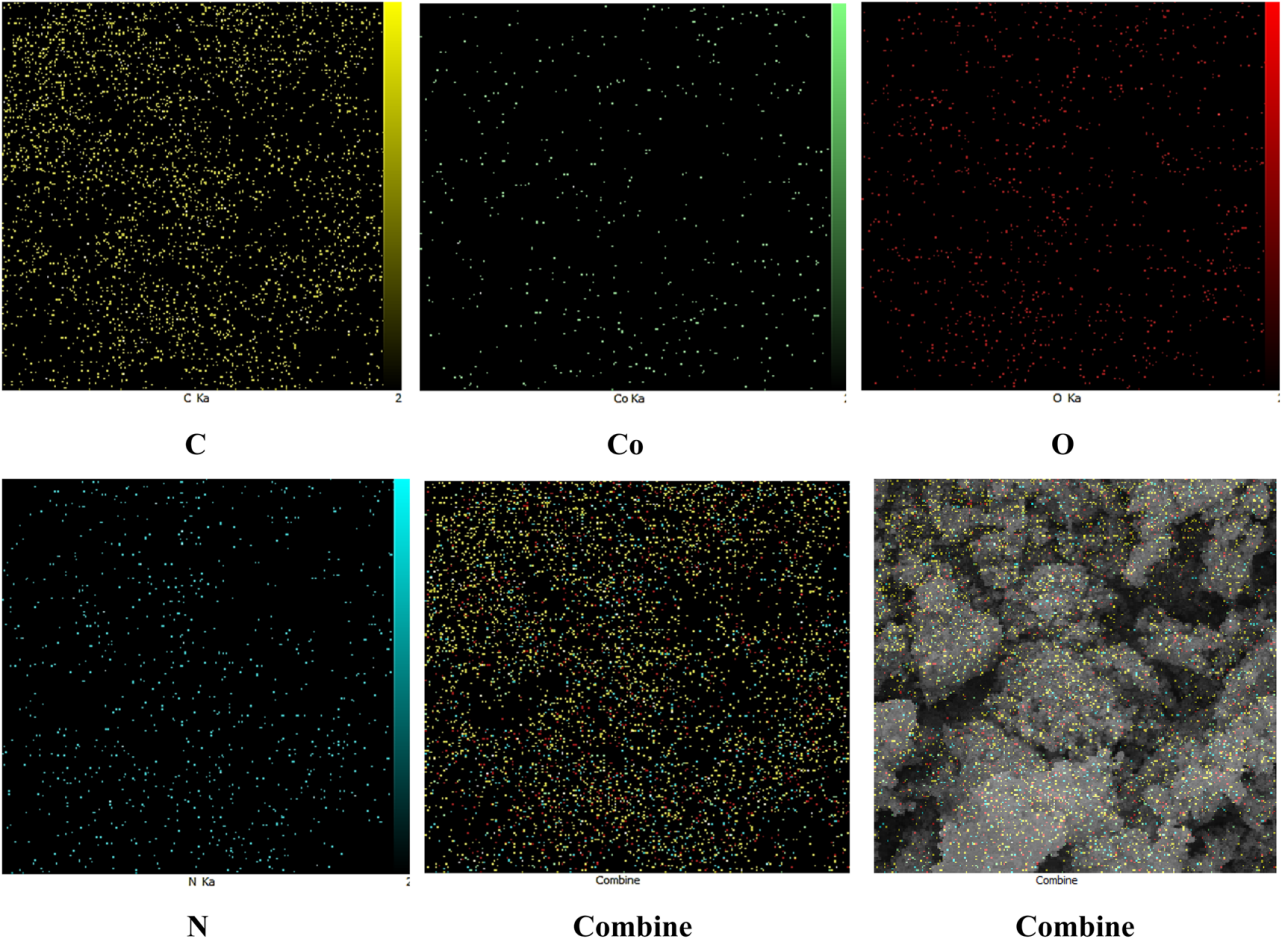
The elemental mapping of the C, N, O, and Co atoms in Co(BDC-NH_2_)–TA–PY.

In elemental mapping analysis, the even distribution of all elements (N, C, O, and Co) within the MOF matrix suggests a thoroughly integrated material characterized by active functional sites. The presence of nitrogen in a well-dispersed manner highlights the role of the triazine–aminopyrimidine moiety as a donor nitrogen ligand, which is critical for enhancing the material's catalytic performance. This attribute establishes Co(BDC-NH_2_)–TA–PY nanocatalyst as a viable option for catalytic applications and the extraction of heavy metals (Fig. 6).

#### 3.1.6. Porosity studies (BET)

**Fig. 7:**
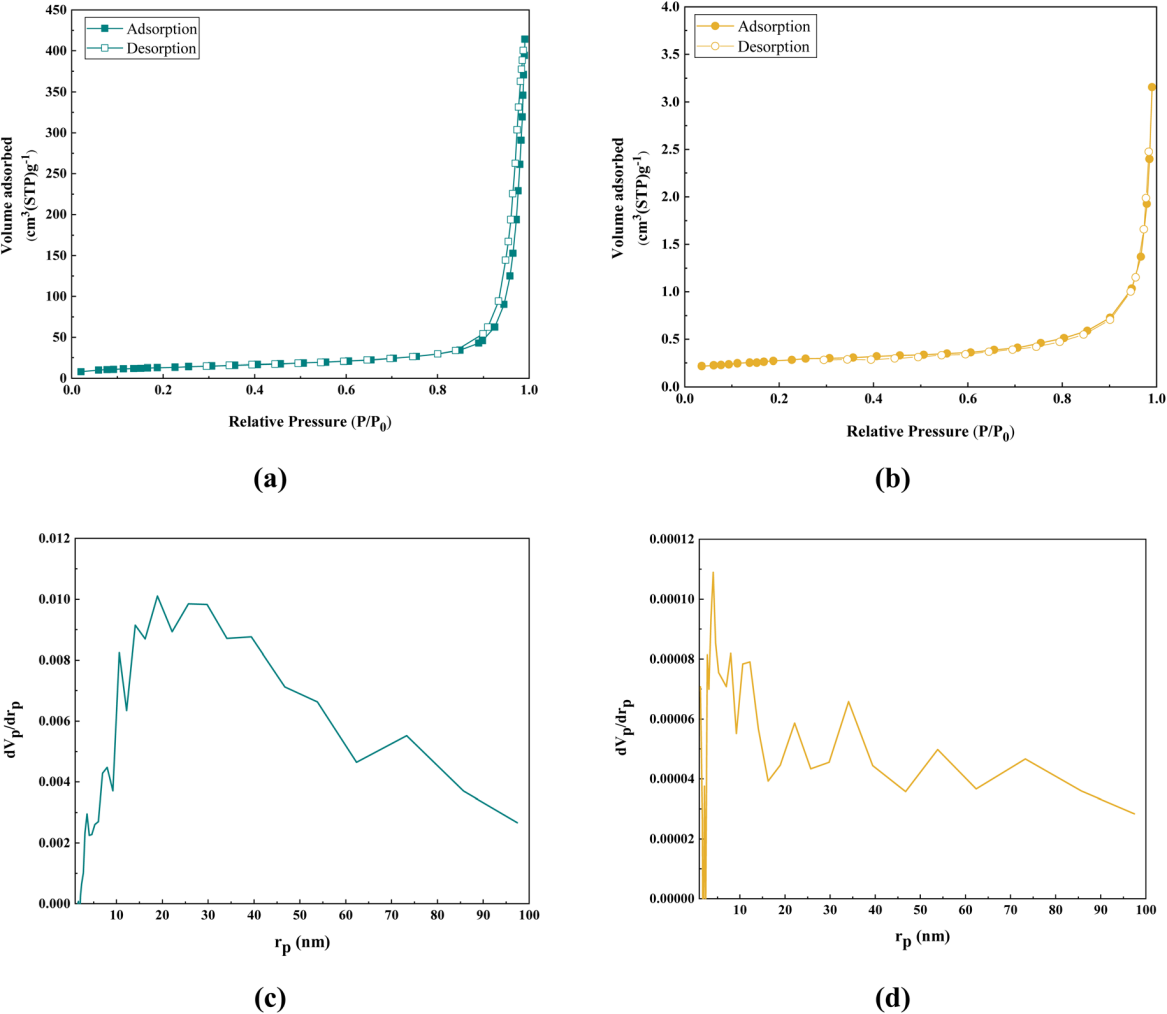
Nitrogen adsorption/desorption isotherms of (a) Co(BDC-NH_2_), (b) Co(BDC-NH_2_)–TA–PY, and BJH diagram of (c) Co(BDC-NH_2_), (d) Co(BDC-NH_2_)–TA–PY.

The synthesized porous Co(BDC-NH_2_) sample exhibited type IV isotherms, characteristic of mesoporous materials, along with type H4 hysteresis loops, indicative of slit-shaped pores (Fig. 7a). Similarly, the Co(BDC-NH_2_)–TA–PY sample displayed type IV isotherms, typical for mesoporous structures, but with type H4 hysteresis loops (Fig. 7b).

The BET surface area of Co(BDC-NH_2_)–TA–PY was measured at 1.30 m^2^ g^−1^, significantly lower compared to Co(BDC-NH_2_), which had a surface area of 64.18 m^2^ g^−1^ (Table 2). The BJH analysis method, also known as Barrett–Joyner–Halenda, is used to study the pore size distribution of porous materials.^37^ The BJH diagrams of Co(BDC-NH_2_) and Co(BDC-NH_2_)–TA–PY are shown in Fig. 7(c and d). According to the diagrams, it can be said that for Co(BDC-NH_2_), most of the pores have a size of 20 to 40 nm, and for Co(BDC-NH_2_)–TA–PY, most of the pores have a size of less than 5 nm. The observed changes in the textural properties of the Co(BDC-NH_2_)–TA–PY nanocatalyst are attributed to the successful immobilization of the triazine–pyrimidine moiety, which likely altered the pore structure and surface characteristics of the material. Grafting of TA–PY on the surface facilitates the blocking of micropores and/or small mesopores through its triazine–amine functionality, which effectively reduces the surface area and pore volume.^38^

**Table 2:** Results of the Langmuir and BET measurements

Parameter	Co(BDC-NH_2_)	Co(BDC-NH_2_)–TA–PY
*a*_s_ (m^2^ g^−1^)	64.181	1.30
*V*_m_ (cm^3^ (STP) g^−1^)	14.746	0.2995
*V*_p_ (cm^3^ g^−1^)	0.6043	0.0045
*r*_p_ (nm)	18.92	4.05

#### 3.1.7. TGA analysis

**Fig. 8:**
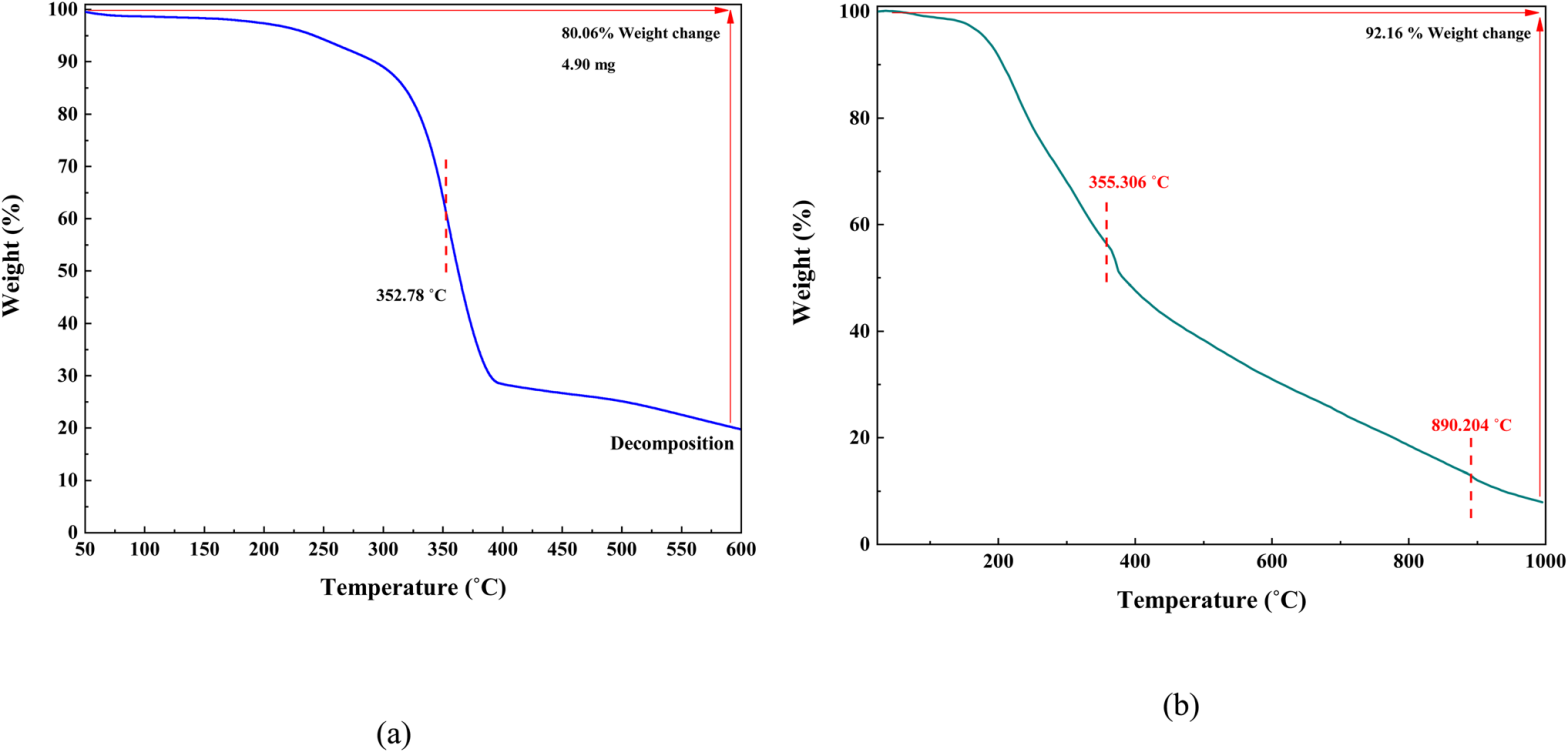
TGA analysis of Co(BDC-NH_2_) (a), and Co(BDC-NH_2_)–TA–PY (b).

TGA plot of Co(BDC-NH_2_) and Co(BDC-NH_2_)–TA–PY reveals a total weight loss of 80.06% and 92.16% over the temperature range of 25–1000 °C (Fig. 8). According to Fig. 8a, Co(BDC-NH_2_) decomposes in one step at 352.78 °C, indicating that the material has high thermal stability.

The TGA profile of Co(BDC-NH_2_)–TA–PY is divided into three distinct weight loss steps, each corresponding to different thermal decomposition processes (Fig. 8b). The initial weight loss at 120–240 °C (∼7%) is attributed to the removal of moisture, trapped organic solvents, and CO_2_ from the nanocatalyst structure. This step is consistent with the evaporation of volatile components and does not affect the structural integrity of the material.^39^ The second weight loss step is associated with the decomposition of the TA–PY organic moieties and 2-aminoterephthalate ligands, confirming the successful functionalization of the Co(BDC-NH_2_) framework with the TA–PY groups. These results indicate that Co(BDC-NH_2_)–TA–PY demonstrates thermal stability up to 335.306 °C. The third step of weight loss is ascribed to the decomposition of the framework, signifying the breakdown of the metal–organic structure, leading to the complete degradation of the material.^40^

### 3.2. Model reaction optimization

The catalytic performance of Co(BDC-NH_2_) and different concentrations of Co(BDC-NH_2_)–TA–PY solids was investigated for the synthesis of triazoles from the reaction of benzaldehydes, nitromethane, and sodium azide. The achieved results are shown in Table 3. A blank control experiment at 100 °C without any catalyst showed no expected product (entry 1). Later, the addition of the Co(BDC-NH_2_) catalyst did not afford any significant expected product after 24 h under similar conditions (entry 2). Meanwhile, the presence of 0.03 g of Co(BDC-NH_2_)–TA–PY catalyst in the medium under identical conditions afforded a 94% yield (entry 4), while the employment of 0.01 g, 0.04 g, and 0.05 g Co(BDC-NH_2_)–TA–PY catalysts gave 86%, 94%, and 94% yields, respectively, under identical conditions (entries 3–5). The catalytic potential was studied using various organic solvents, including ethanol, water, solvent-free, dimethylformamide (DMF), and toluene (entries 6–12). Notably, the model reaction achieved the highest yield in dimethylformamide (entry 4). Additionally, when the reaction temperature was lowered to 80 °C, the product yield dropped to 84% under the same reaction conditions (entry 13). Furthermore, when the temperature increased to 120 °C, the yield decreased to 92% (entry 14). Finally, we conducted a comparison of the efficiency between Co(BDC-NH_2_)–TA–PY nanocatalyst, triazine, and 2-aminopyrimidine groups. The best result was obtained when the Co(BDC-NH_2_)–TA–PY nanocatalyst was utilized, as shown in Table 3, entries 15 and 16. This observation emphasizes the distinctive catalytic efficiency of Co(BDC-NH_2_)–TA–PY in facilitating this particular organic transformation. By providing a platform for adsorption and reaction sites, the functionalized Co(BDC-NH_2_) facilitates the conversion of reactants into desired products with enhanced efficiency. Moreover, Co(BDC-NH_2_)–TA–PY accelerates the reaction by activating the reactants. After purification of products, the purified product was weighed, and the reaction yield was calculated using the simple formula (mass of purified product ÷ theoretical mass × 100). The reported yields are isolated yields.

**Table 3:** Screening the reaction parameters for the synthesis of triazoles using Co(BDC-NH_2_)–TA–PY^a^

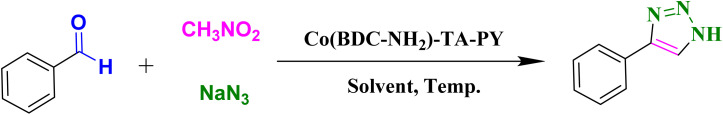					
Entry	Catalyst (mg)	Solvent	Temperature (°C)	Time (h)	Yield^b^ (%)
1	—	DMF	100	1	Trace
2	Co(BDC-NH_2_) (30 mg)	DMF	100	24	42
3	10	DMF	100	1	86
**4**	**30**	**DMF**	**100**	**1**	**94**
5	40	DMF	100	1	94
6	50	DMF	100	1	94
7	30	Solvent-free	100	1	Trace
8	30	H_2_O	Reflux	1	N.R.
9	30	Ethanol	Reflux	1	N.R.
10	30	PEG	Reflux	1	Trace
11	30	Acetonitrile	Reflux	1	Trace
12	30	Toluene	Reflux	1	N.R.
13	30	DMF	80	1	84
14	30	DMF	120	1	92
15	Triazine (1 mmol)	DMF	100	24	30
16	2-Aminopyrimidine (1 mmol)	DMF	100	24	28

a Reaction conditions: aldehyde (1 mmol), CH_3_NO_2_ (1 mmol), NaN_3_ (1 mmol), solvent (5 mL), in the presence of Co(BDC-NH_2_)–TA–PY.

b Isolated yield.

Subsequently, the optimized conditions were established, and the stability of the catalyst was confirmed, leading to an evaluation of the substrate scope of various substituted 4-aryl-NH-1,2,3-triazoles with benzaldehyde derivatives utilizing a Co(BDC-NH_2_)–TA–PY solid catalyst (Table 4).^41–43^ The reaction was carried out with electron-withdrawing, electron-donating, heterocyclic benzaldehyde, and terephthalaldehyde. Table 4 presents a concise overview of the observed outcomes. Initially, the benzaldehyde substrate reacted with sodium azide and nitromethane and yielded 92% (4a) under optimal reaction conditions. Comparable yields of 95% and 90% (4b and 4c) were achieved by the benzaldehyde substituents with 4-methoxy and 4-methyl groups, respectively. Furthermore, the substitution of 2-methoxy and *N*,*N*-dimethyl on the benzaldehyde resulted in successful reactions, producing 4-aryl-NH-1,2,3-triazoles at 92% and 91% yields (4d and 4e), respectively. Moreover, benzaldehydes with *para*-substituted electron-withdrawing groups such as –Cl, –Br, and –NO_2_ yielded desirable products at 93%, 88%, and 91% yields (4f, 4g and 4h), respectively. Additionally, upon substituting phenyl with *m*-NO_2_, the reaction progressed efficiently, yielding desirable products at 94% yields (4i). Benzaldehyde derivatives with electron-withdrawing groups achieved high efficiency in a shorter time than benzaldehydes with electron-donating groups. Furthermore, indole-3-carbaldehyde, furfural, and terephthalaldehyde gave 85%, 88%, and 76% yields (4j, 4k and 4l), respectively.

**Table 4:** Synthesis of various 1,2,3-triazole derivatives in the presence of catalyst^a^

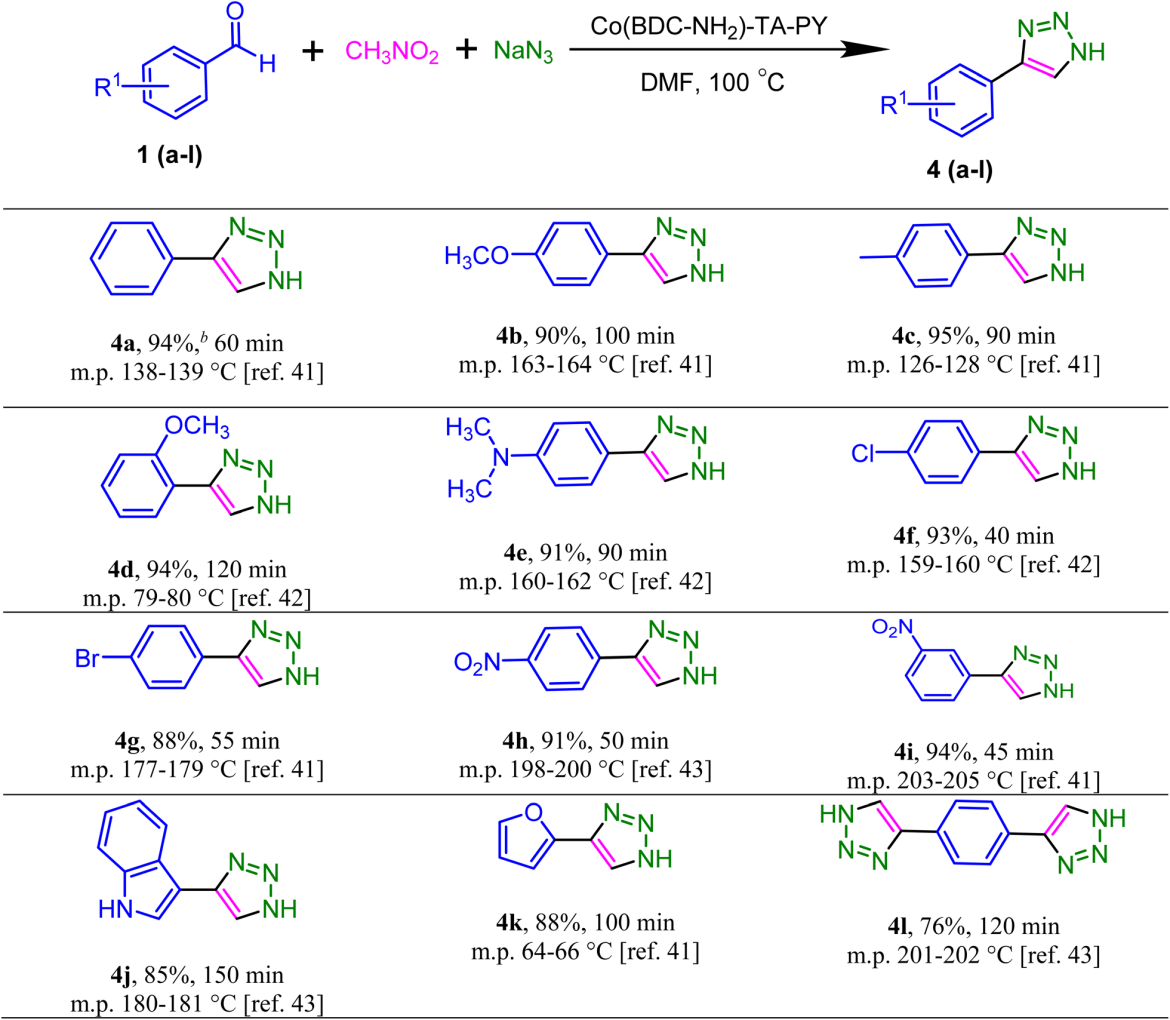

a Reaction conditions: benzaldehyde derivatives (1.0 mmol), nitromethane (1.0 mmol), sodium azide (1 mmol), and Co(BDC-NH_2_)–TA–PY (30 mg) was heated in DMF (2 mL) at 100 °C.

b Based on isolated yield.

### 3.3. Reaction mechanism

**Scheme 4:**
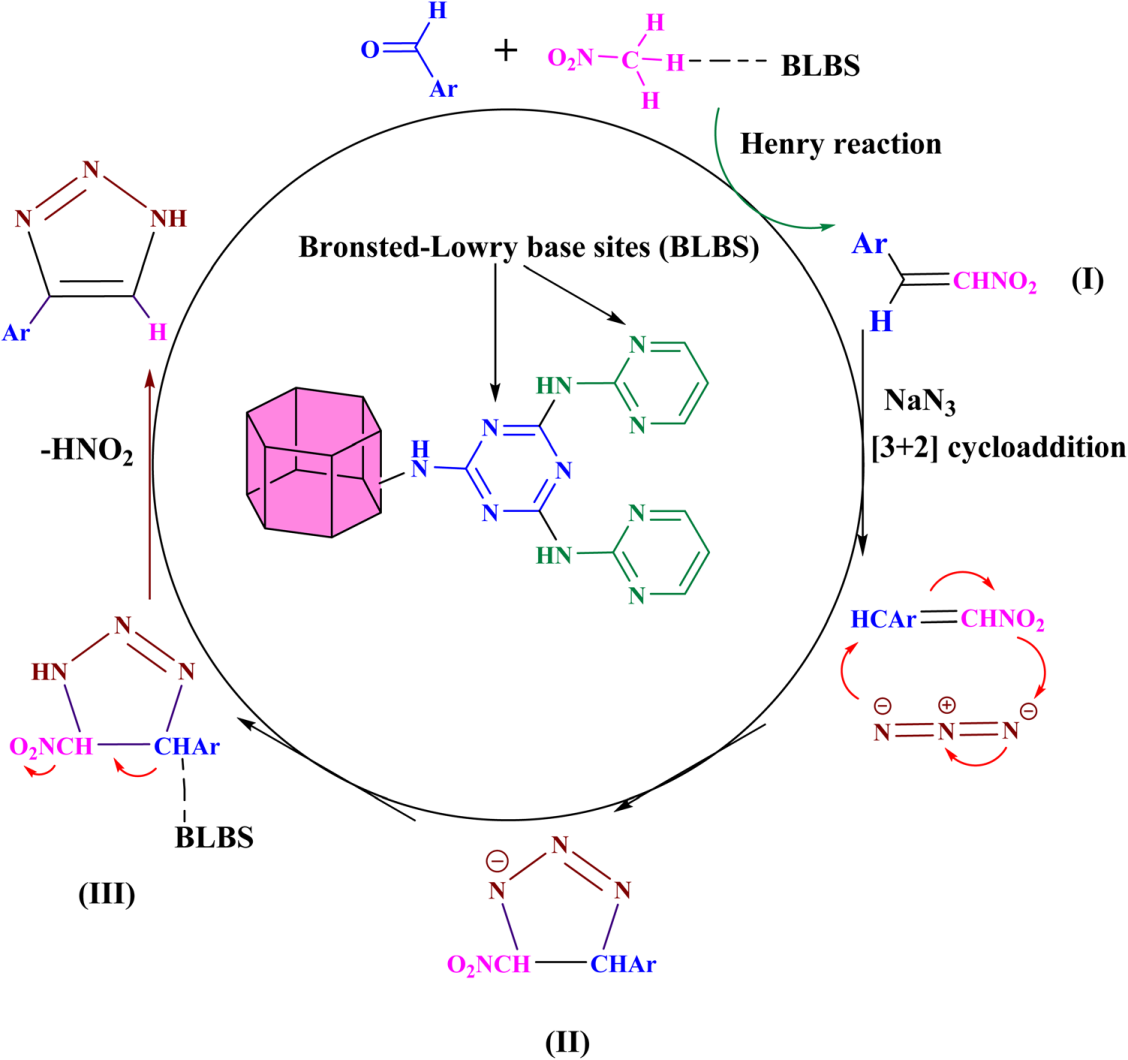
The proposed synthesis mechanism of 4-aryl-NH-1,2,3-triazoles in the presence of Co(BDC-NH_2_)–TA–PY.

The synthesis of 4-aryl-NH-1,2,3-triazoles using Co(BDC-NH_2_)–TA–PY as a solid catalyst with Brønsted–Lowry Base Sites (BLBS) can be proposed to proceed through a two-step mechanism: the Henry reaction followed by a [3 + 2] cycloaddition. The Brønsted–Lowry Base Sites (BLBS) in Co(BDC-NH_2_)–TA–PY deprotonate nitromethane, generating a nitronate anion. The nitronate anion then undergoes nucleophilic addition to an activated aldehyde, forming a β-nitro alcohol intermediate. The β-nitro alcohol intermediate undergoes dehydration (elimination of water), leading to the formation of a nitroalkene intermediate (I). Intermediate (I) then undergoes nucleophilic addition with sodium azide, which is subsequently followed by a [3 + 2] cycloaddition to yield the cyclized intermediate II. Intermediate (II) is further activated by Co(BDC-NH_2_)–TA–PY, converting it into intermediate (III). Finally, Co(BDC-NH_2_)–TA–PY oxidizes intermediate (III) further to triazoles with the elimination of HNO_2_ (Scheme 4).^44^

**Fig. 9:**
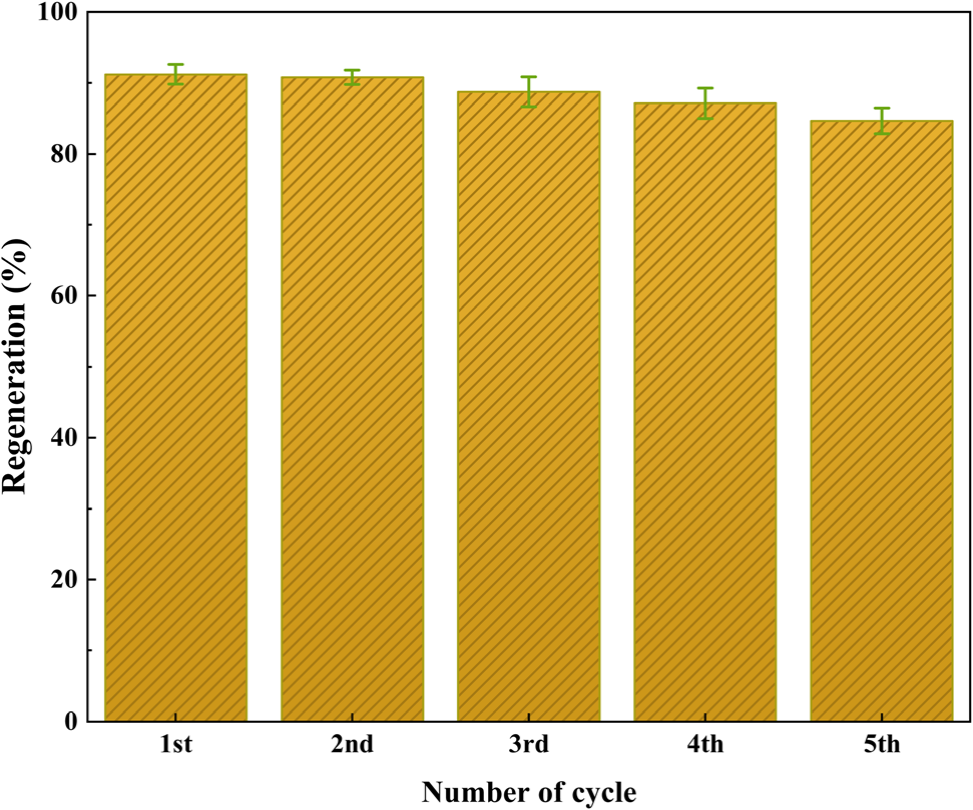
Reusability data of Co(BDC-NH_2_)–TA–PY nanocatalyst.

One of the main objectives of developing Co(BDC-NH_2_)–TA–PY solid heterogeneous catalyst is to recover and reuse the catalyst. In this aspect, the performance of Co(BDC-NH_2_)–TA–PY under the optimized reaction conditions was used in consecutive cycles, and the observed results are shown in Fig. 9. Notably, the study revealed admirable catalytic activity over five reaction cycles, with slightly decreased yield after five reaction cycles. After each cycle, the catalyst was washed with ethanol several times, centrifuged, and dried at 100 °C for 8 h. This solid was reused in subsequent runs of the model reaction.

**Fig. 10:**
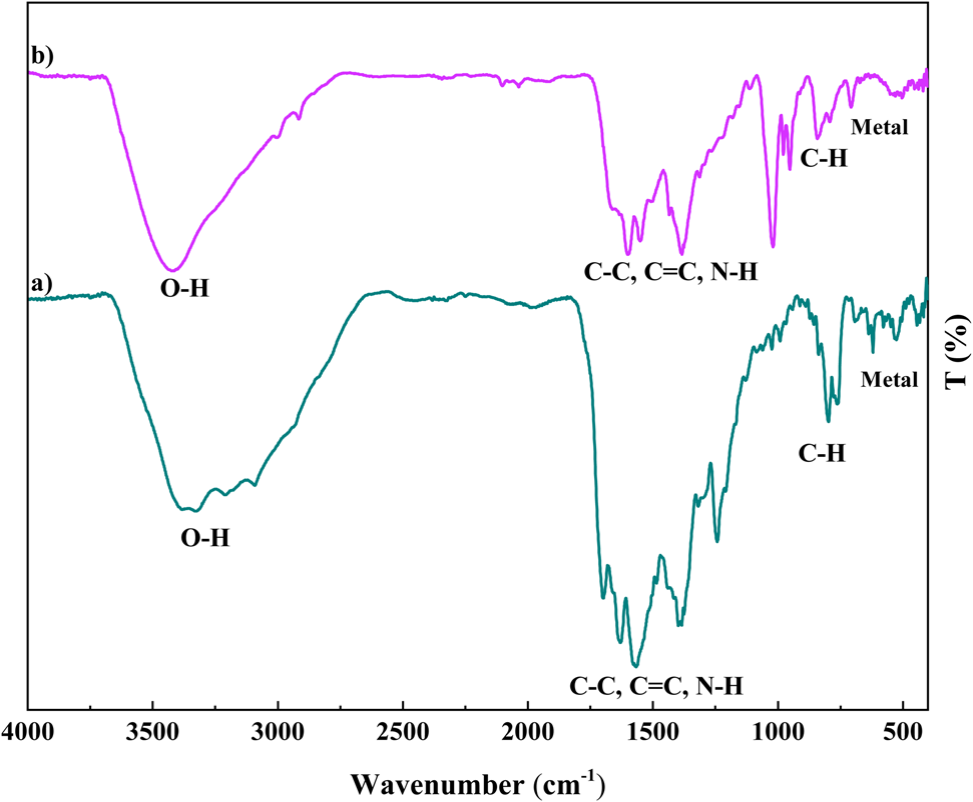
FTIR analysis of fresh (a) and recycled (b) Co(BDC-NH_2_)–TA–PY nanocatalyst.

**Fig. 11:**
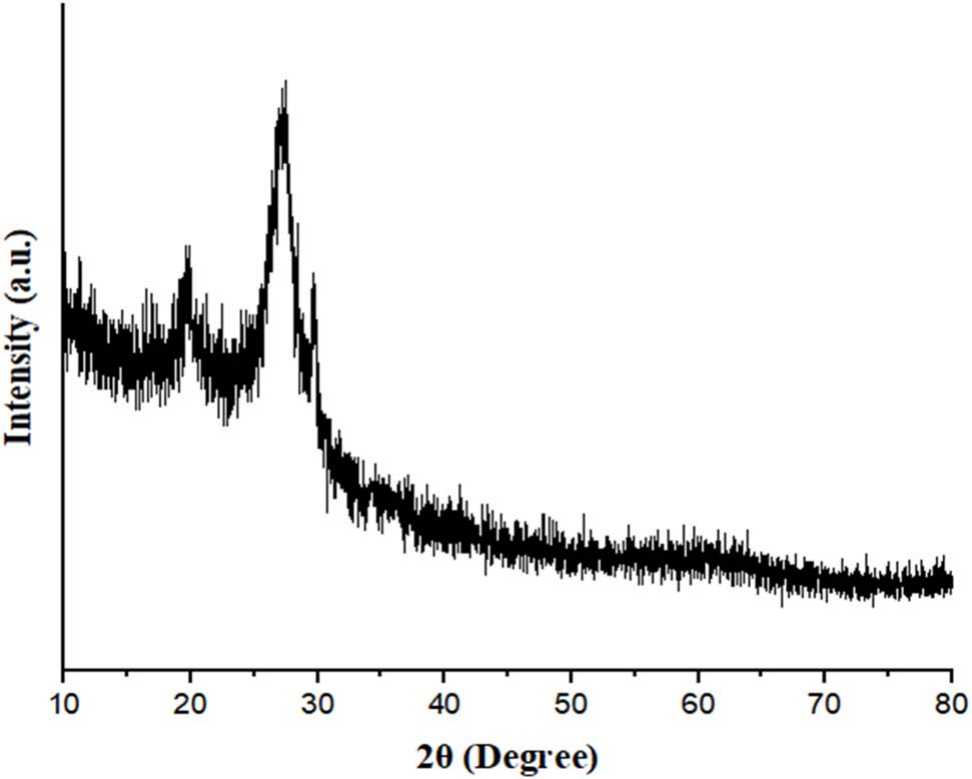
XRD analysis of recycled Co(BDC-NH_2_)–TA–PY nanocatalyst.

**Fig. 12:**
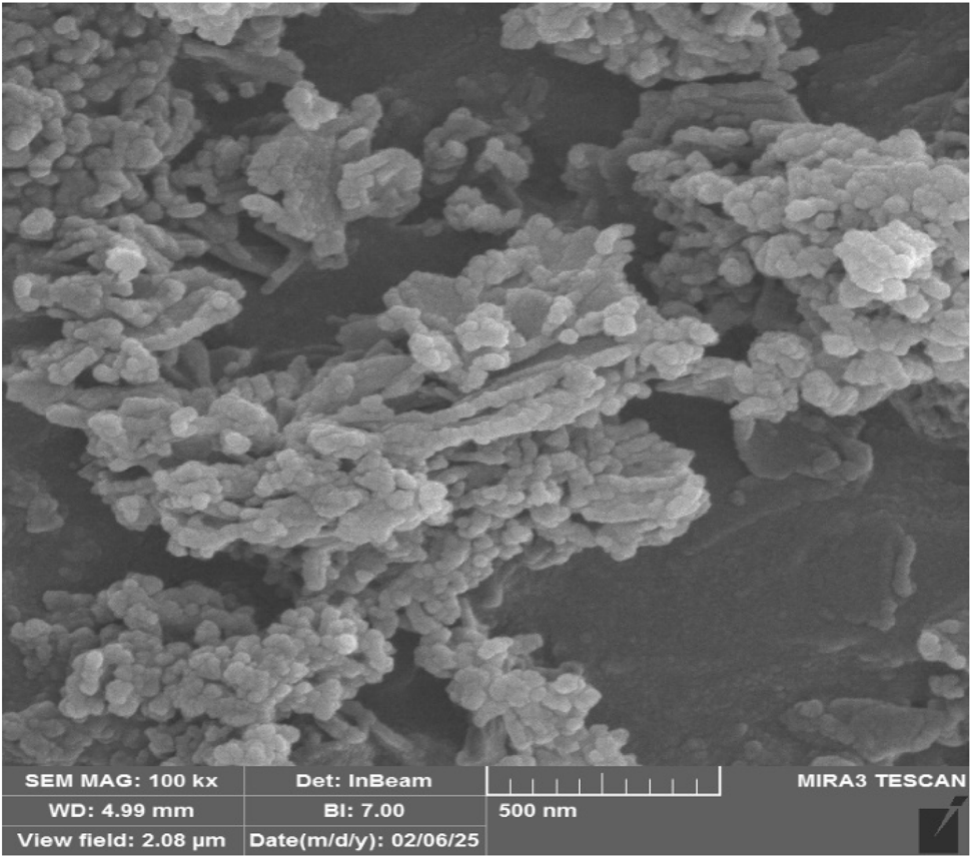
FESEM analysis of recycled Co(BDC-NH_2_)–TA–PY.

The chemical and surface structure stability of the catalyst after recycle experiments were analyzed using FT-IR (Fig. 10), XRD analysis (Fig. 11), and FESEM measurements (Fig. 12). These results indicate that the structural integrity, crystallinity, and morphology of the reused catalyst remained unchanged, demonstrating its good stability.

### 3.4. Hot filtration test

**Fig. 13:**
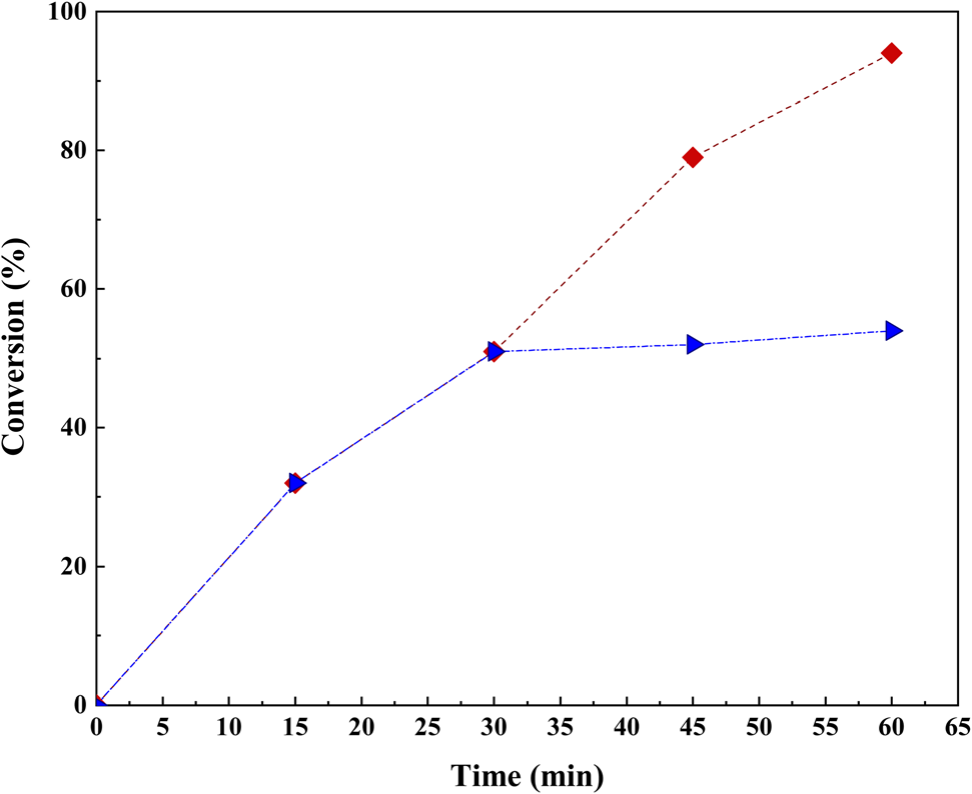
Hot-filtration test for the synthesis of 4a.

To investigate the homogeneous or heterogeneous nature of the Co(BDC-NH_2_)–TA–PY nanocomposite as a catalyst in the model reaction, a hot filtration test was conducted as a reliable method. Initially, a mixture of Co(BDC-NH_2_)–TA–PY nanocomposites (30 mg) and DMF (2 mL) was stirred at 100 °C for 30 minutes. Subsequently, the reaction mixture was filtered, and the catalyst was separated from the solution. Following this, a mixture of benzaldehyde (1.0 mmol), nitromethane (1.0 mmol), sodium azide (1 mmol), and filtrate solvent (2 mL) was prepared and stirred at 100 °C for 30 minutes. Investigation of the reaction progress using TLC revealed a negligible yield of 51%. This indicates a lack of significant reaction progress and the heterogeneous nature of the catalyst used. The obtained results confirm that the reaction progress is directly dependent on the presence of the catalyst, and the filtration process completely separated the catalyst from the reaction mixture. Therefore, the leaching phenomenon was not observed in this system (Fig. 13).^45,46^ The concentration of cobalt was determined by ICP-AES, which changed from 0.53 mmol g^−1^ to 0.52 mmol g^−1^ after the 5th run. Therefore, the Co(BDC-NH_2_)–TA–PY is highly stable under the studied reaction conditions.

We compared the current method for synthesizing triazole with reactions conducted using Ni(OH)_2_–ZnO,^47^ AlCl_3_,^48^*p*-TsOH,^49^ and piperidine-sulfated zirconia^50^ (Table 5). All these methods require harsh reaction conditions and longer reaction times compared to our present approach. The synthesis reaction is completed in a significantly shorter time compared to other systems. The process operates under milder conditions, reducing energy consumption and potential side reactions. The catalyst can be easily separated from the reaction mixture, facilitating product isolation. The synthesis of Co(BDC-NH_2_)–TA–PY nanocatalyst is straightforward and reproducible from available starting materials (entry 5). These advantages collectively position the Co(BDC-NH_2_)–TA–PY catalyst as a highly effective and practical option for synthesizing 4-aryl-NH-1,2,3-triazoles, outperforming existing catalytic systems in terms of efficiency, convenience, and sustainability.

**Table 5:** Comparison of the present catalytic data with earlier reported procedure for synthesis 4a product

Entry	Catalyst	Conditions	Yield (%)	Time	Ref.
1	Ni(OH)_2_–ZnO	PEG-400, 100 °C	65–96	3.5–5 h	47
2	AlCl_3_	DMSO, MW, 70 °C	59–95	24 h	48
3	*p*-TsOH	DMF, 60 °C	66–97	1–3 h	49
4	Piperidine-sulfated zirconia	DMF, MW, 80 °C	85–92	30 min	50
5	Co(BDC-NH_2_)–TA–PY	DMF, 100 °C	76–94	1–2 h	This work

## 4. Conclusion

This study successfully demonstrated the synthesis of a novel, recyclable, and environmentally friendly catalyst, Co(BDC-NH_2_)–TA–PY, through post-synthetic modification of Co(BDC-NH_2_) with novel nitrogen-rich moieties (triazine–pyrimidine (TA–PY) functional groups). The integration of TA–PY ligands into the cobalt-based metal–organic framework significantly enhanced its catalytic activity. This bifunctional catalyst exhibited remarkable efficiency in synthesizing triazoles *via* the Henry condensation followed by a [3 + 2] cycloaddition reaction under mild conditions. The characteristic properties such as functionality, crystallinity, morphology, and thermal stability of the nanocatalyst were well scrutinized using various characterization techniques and found to be highly significant. A range of novel substituted triazoles was synthesized in high yields in a short reaction time under optimized reaction conditions. The remarkable outcomes of the methodology are a simple, environmentally friendly, recyclable, and heterogeneous nanocatalyst. Future research could explore extending this approach to other MOF systems and catalytic reactions, further expanding the scope of these materials in industrial and pharmaceutical processes.

## Conflicts of interest

There are no conflicts to declare.

## Supplementary Material

NA-007-D5NA00299K-s001

## Data Availability

Data is provided within the manuscript or ESI.†
