# Supplementary material for: Triazoles synthesis using nanocatalyst triazine–pyrimidine-modified cobalt-based metal–organic frameworks
Source: Nanoscale Adv. 2025 Jul 21;7(17):5346–60. doi: 10.1039/d5na00299k (PMC12301849; doi:10.1039/d5na00299k)

Supplementary Information (SI) for Nanoscale Advances.
This journal is © The Royal Society of Chemistry 2025

Supporting Information for:

Triazoles synthesis using nanocatalyst triazine-pyrimidine-modified cobalt-based metal-organic frameworks

Mahtab Amirian[a], Ramin Ghorbani-Vaghei*[,a,b], Sedigheh Alavinia[a]

[a]*Department of Organic Chemistry, Faculty of Chemistry and Petroleum Sciences, Bu-Ali Sina University, 6517838683, Hamadan, Iran. E-mail: rgvaghei@yahoo.com; ghorbani@basu.ac.ir.*

[b]*Department of Organic Chemistry, Faculty of Chemistry, University of Guilan, Rasht, Iran*

4-(4-Chlorophenyl)-1H-1,2,3-triazole:

Melting point:159-160°C

FT-IR (KBr, ν, cm^{-1}): 3159(N-H stretch), 3124(C-H), 1491,1674(C=C), 1588(N-H bending), 1100(C-N), 739(C-Cl)

^{1}H NMR (500 MHz, DMSO-d_6) δ 8.36 (s, 1H), 7.89–7.87 (d, J = 8.5 Hz, 2H), 7.51–7.49 (d, J = 8.5 Hz, 2H).
^{13}C NMR (126 MHz, DMSO) δ 144.35, 132.41, 129.46, 128.85, 127.23, 127.02.

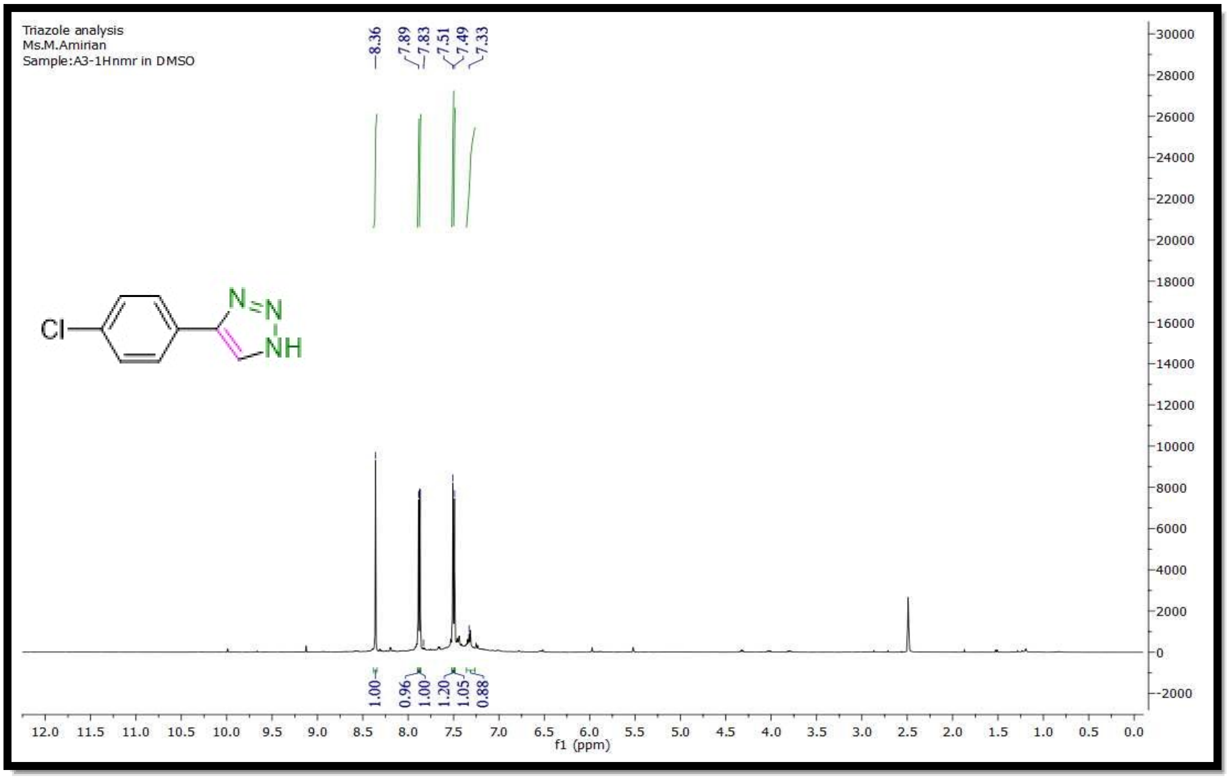

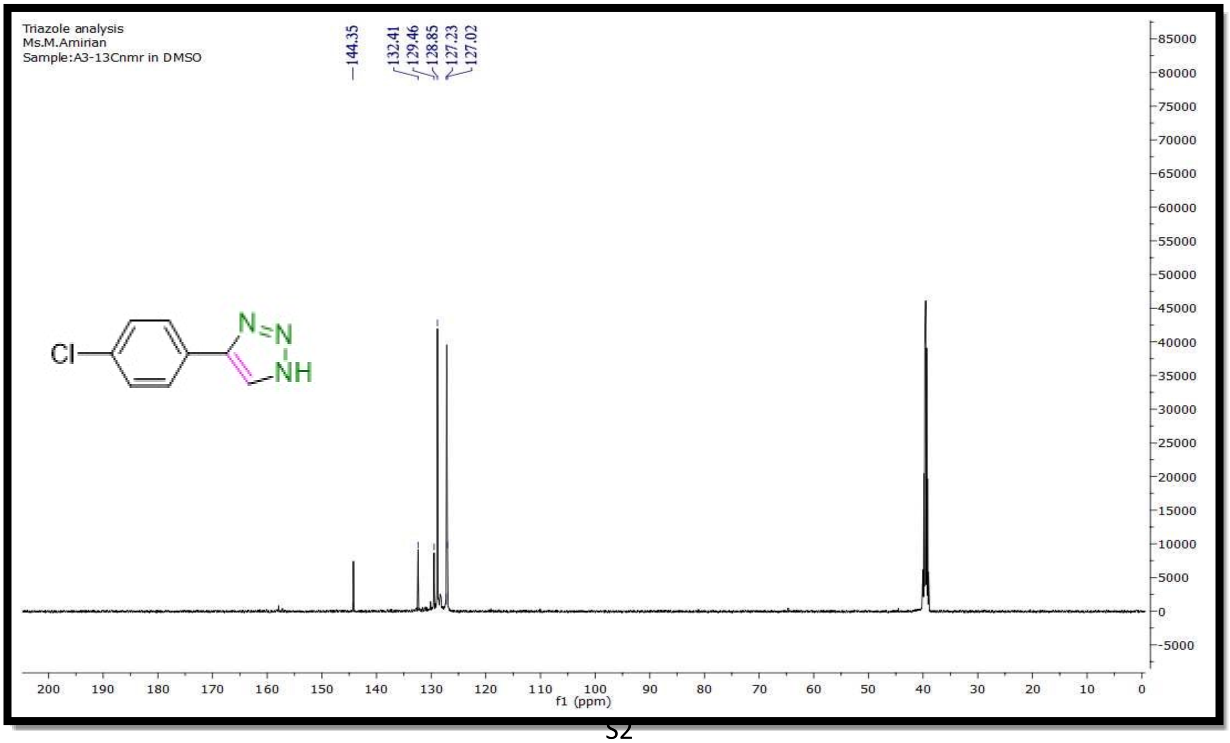

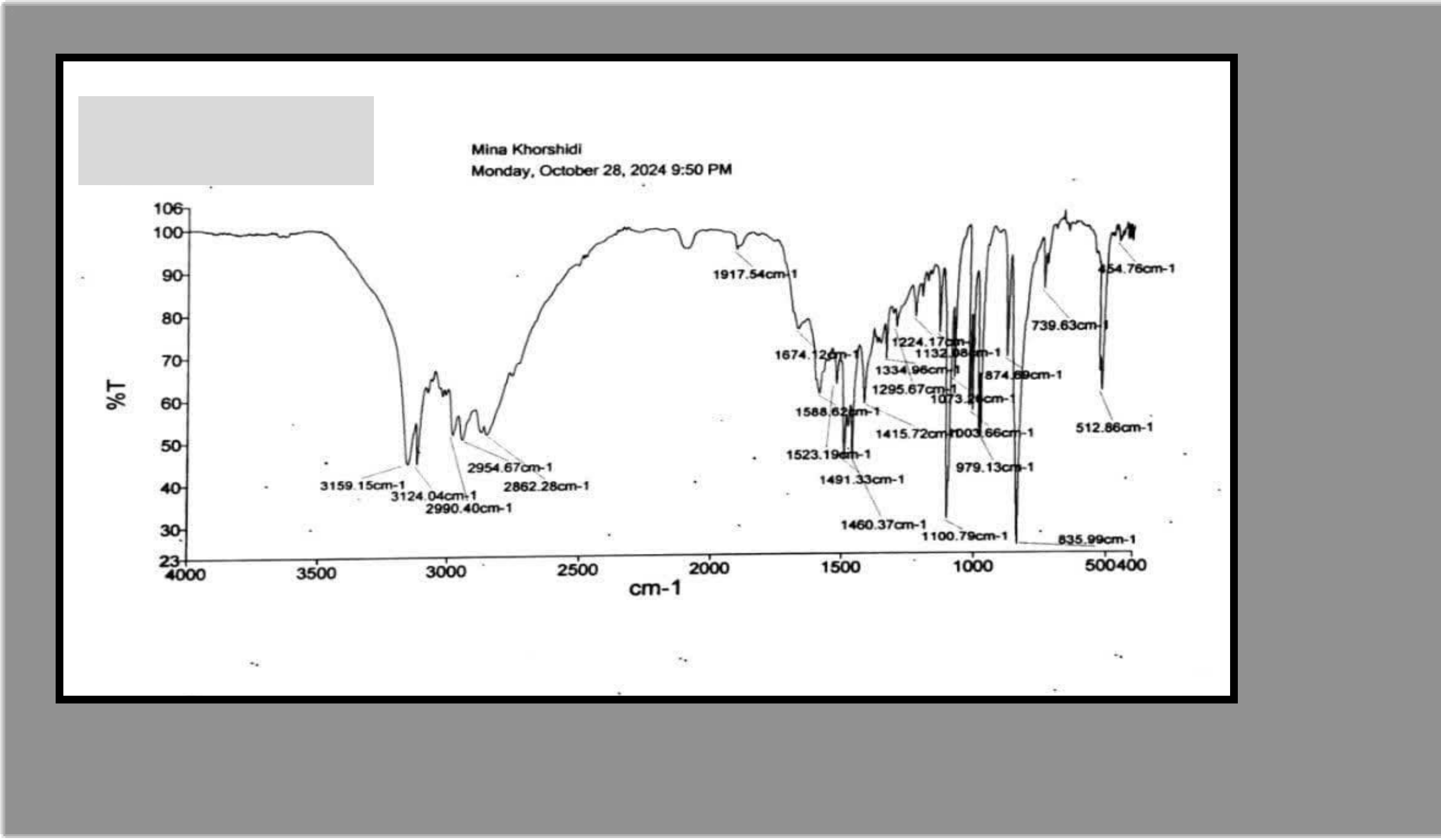

Mina Khorshidi
Monday, October 28, 2024 9:50 PM
%T
cm-1
106
100
90
80
70
60
50
40
30
23
4000
3500
3000
2500
2000
1500
1000
500400
1917.54cm-1
1674.12cm-1
1588.62cm-1
1523.19cm-1
1491.33cm-1
1460.37cm-1
1415.72cm-1
1334.96cm-1
1295.67cm-1
1224.17cm-1
1132.08cm-1
1100.79cm-1
1073.26cm-1
1003.66cm-1
979.13cm-1
874.69cm-1
835.99cm-1
739.63cm-1
512.86cm-1
454.76cm-1
3159.15cm-1
3124.04cm-1
2990.40cm-1
2954.67cm-1
2862.28cm-1

4-(4-Bromophenyl)-1H-1,2,3-triazole:
Melting point: 177-179 °C

FT-IR (KBr, ν, cm^{-1}): 3368(N-H Stretch), 3135(C-H), 1487,1586(C=C), 1010(C-N), 510(C-Br)

^{1}H NMR (500 MHz, DMSO-d_6) δ 8.38 (s, 1H), 7.81-7.83 (s, 2H), 7.63-465 (d, *J* = 8.5 Hz, 3H).
^{13}C NMR (126 MHz, DMSO-d_6) δ 144.29, 132.21, 131.78, 129.75, 127.47, 120.97.

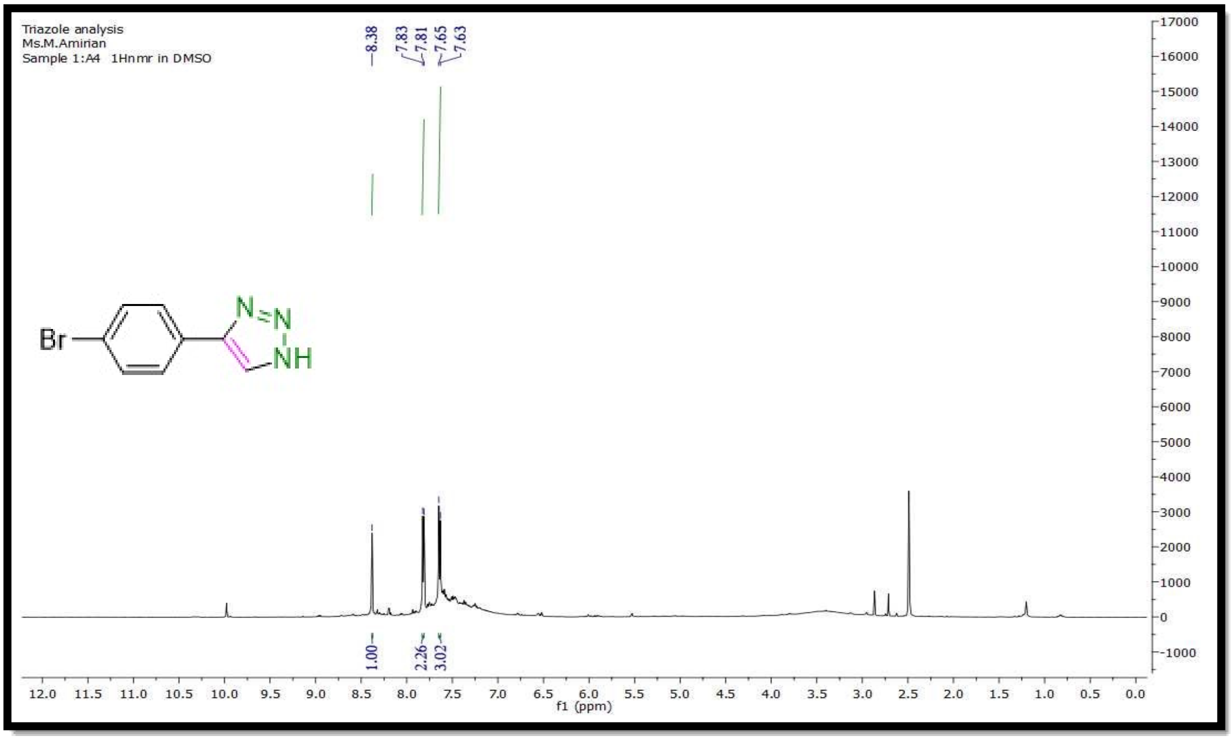

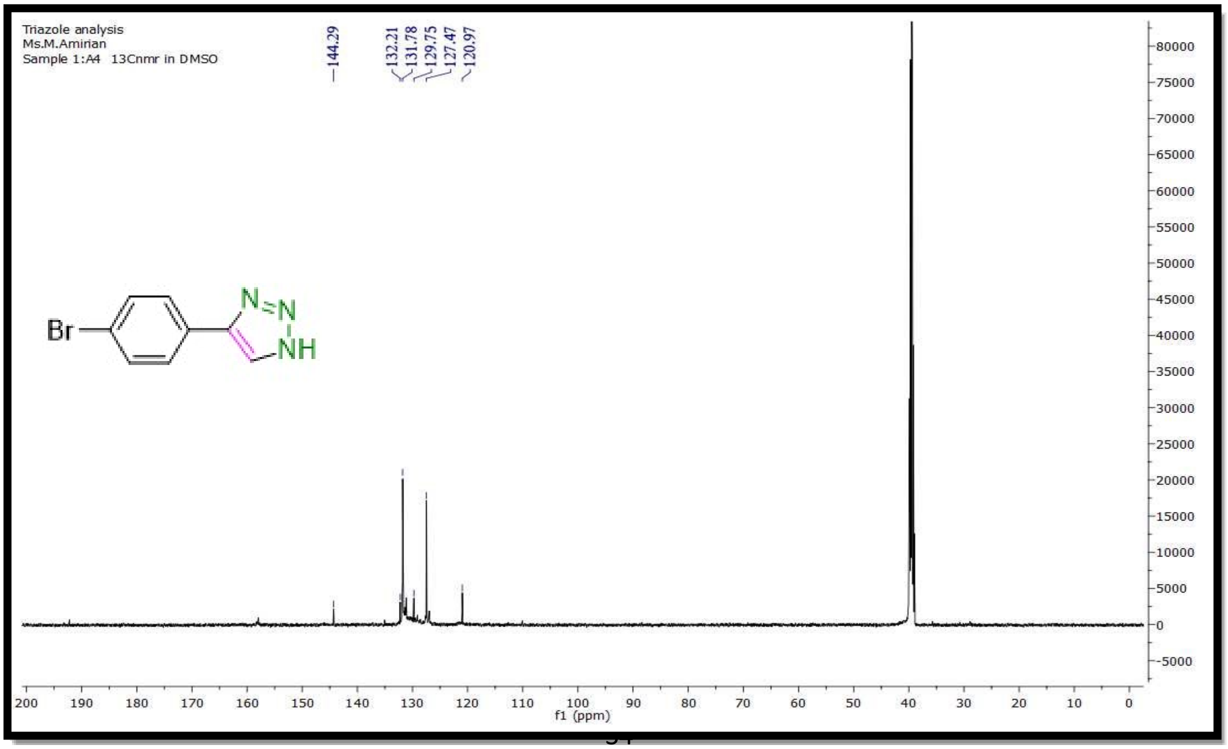

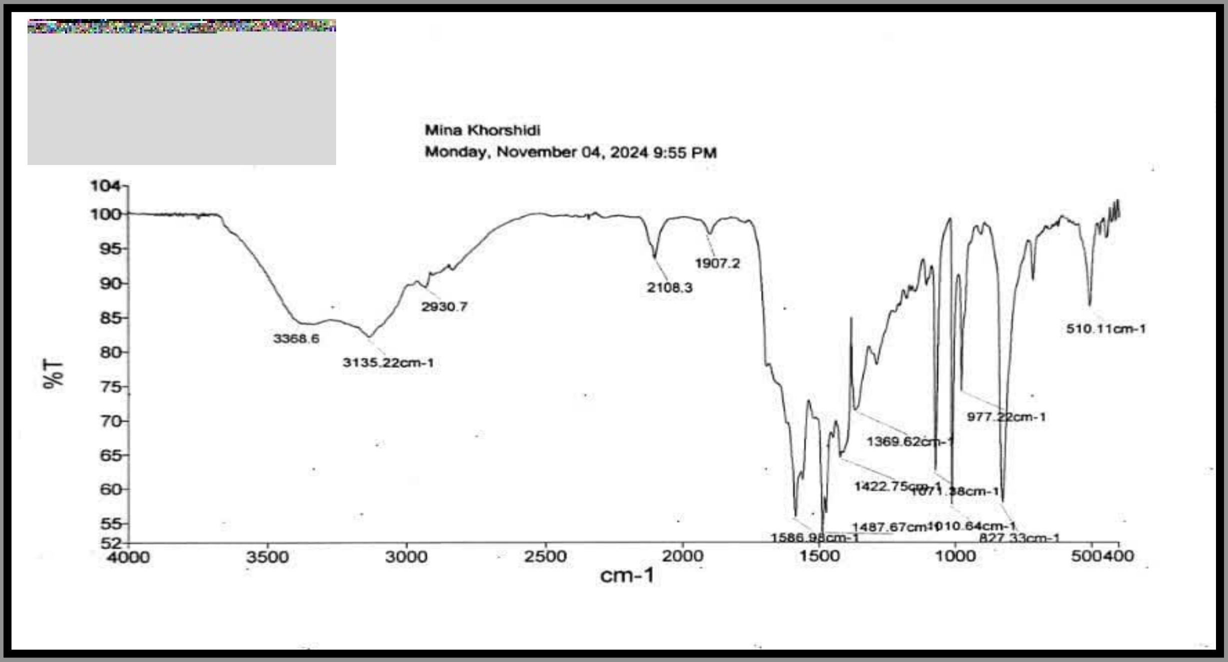

Mina Khorshidi
Monday, November 04, 2024 9:55 PM
%T
cm-1
3368.6
3135.22cm-1
2930.7
2108.3
1907.2
510.11cm-1
977.22cm-1
1369.62cm-1
1422.75cm-1
1071.38cm-1
1487.67cm-1
1010.64cm-1
1586.98cm-1
827.33cm-1

3-(1H-1,2,3-triazol-4-yl)-1H-indole:

Melting point: 180-181 °C

FT-IR (KBr, ν, cm−1): 3170 (N-H Stretch), 3114,3045(C-H), 1446,1635(C=C), 1614(N-H bending), 1244 (C-N)

^{1}H NMR (500 MHz, DMSO) δ 12.14 (s, 1H), 9.94 (s, 1H), 8.27 (s, 1H), 8.09-8.10 (d, *J* = 7.4 Hz, 1H), 7.50-7.52 (d, *J* = 7.4 Hz, 1H), 7.30 – 7.19 (m, 2H).

^{13}C NMR (126 MHz, DMSO) δ 138.14, 137.04, 124.13, 123.38, 122.04, 120.77, 118.17, 112.36.

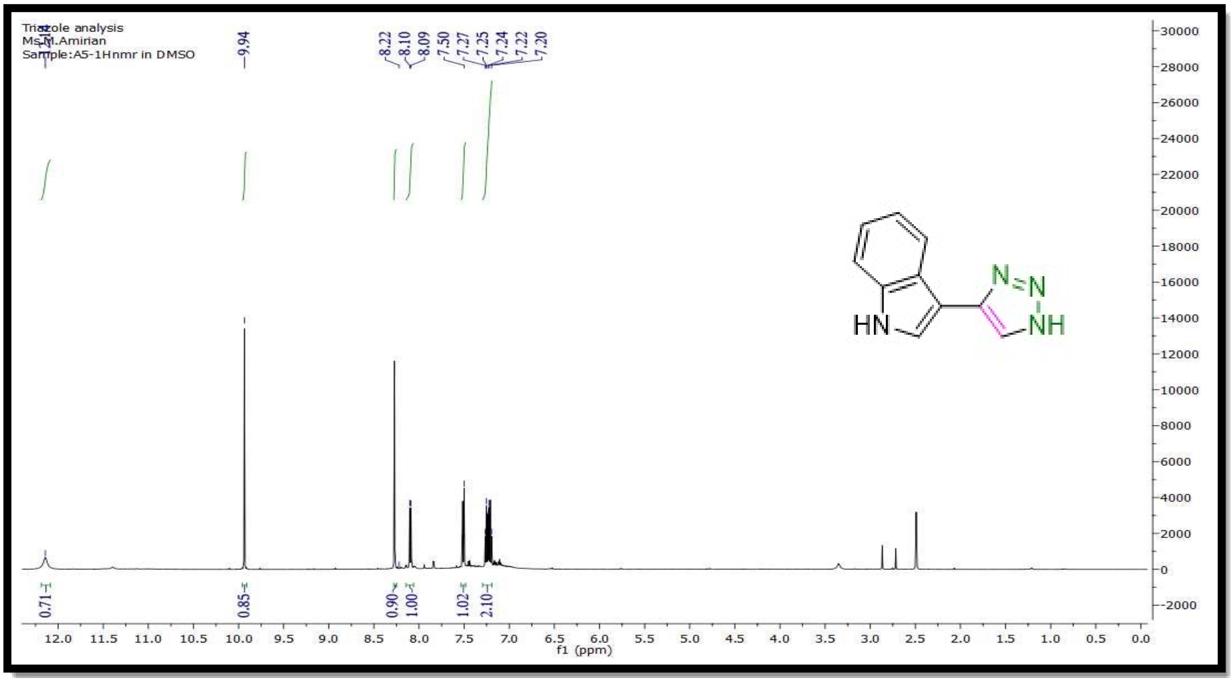

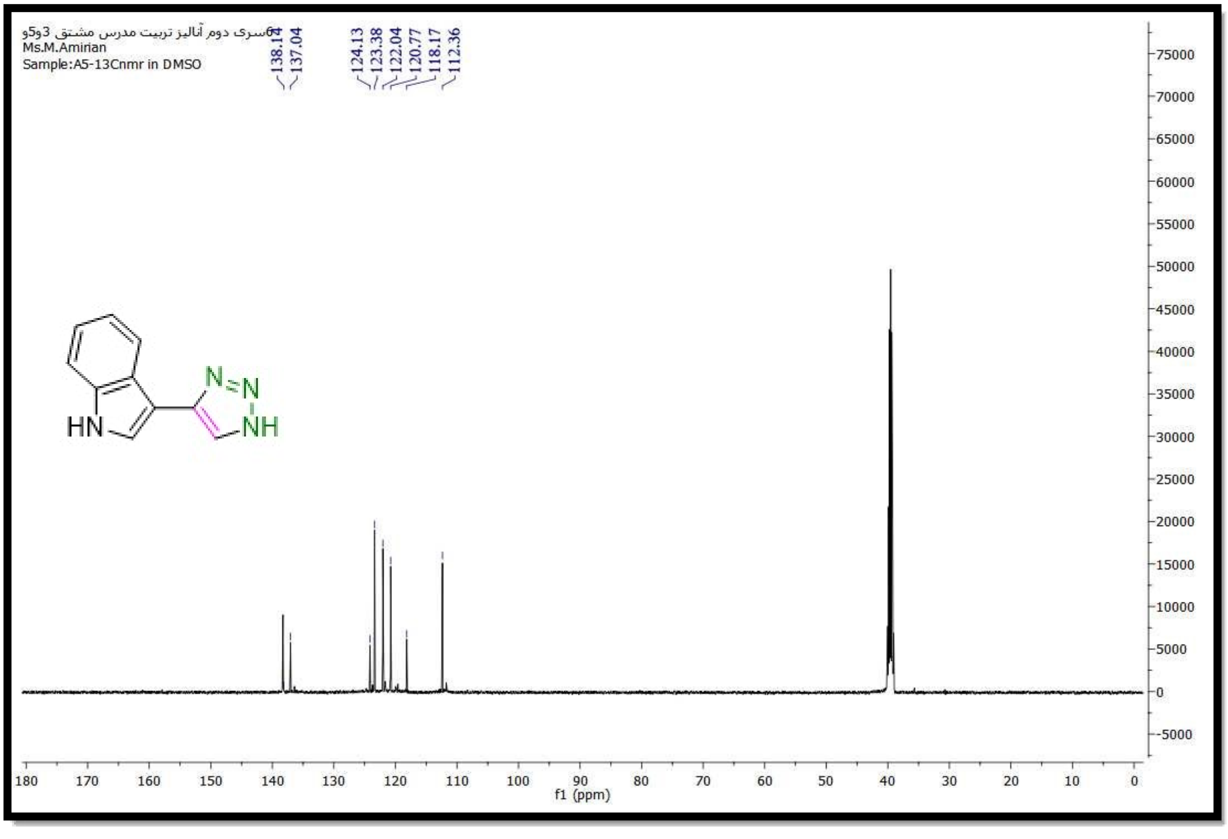

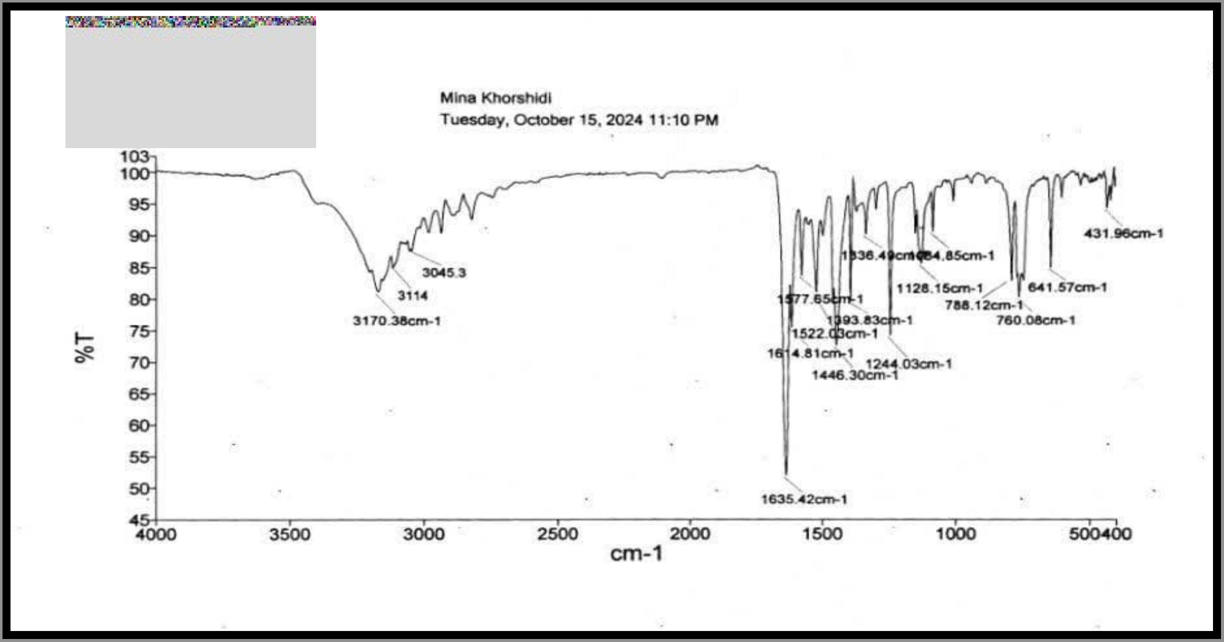
Mina Khorshidi
Tuesday, October 15, 2024 11:10 PM
%T
cm-1
3170.38cm-1
3114
3045.3
1635.42cm-1
1614.81cm-1
1577.65cm-1
1522.03cm-1
1446.30cm-1
1393.83cm-1
1244.03cm-1
1128.15cm-1
788.12cm-1
760.08cm-1
641.57cm-1
431.96cm-1

4-Phenyl-1H-1,2,3-triazole:

Melting point: 138-139 °C

FT-IR (KBr, ν, cm^{-1}): 3160(N-H Stretch), 3116, 2961(C-H), 1454,1657(C=C), 1083(C-N),

^{1}H NMR (500 MHz, DMSO) δ 8.33 (s, 1H), 7.85-7.87 (d, *J* = 7.1 Hz, 2H), 7.43-7.45 (t, 7.7 Hz, 3H), 7.34-7.36 (s, 1H).

^{13}C NMR (126 MHz, DMSO) δ 130.29, 128.84, 128.67, 128.36, 128.02, 125.52.

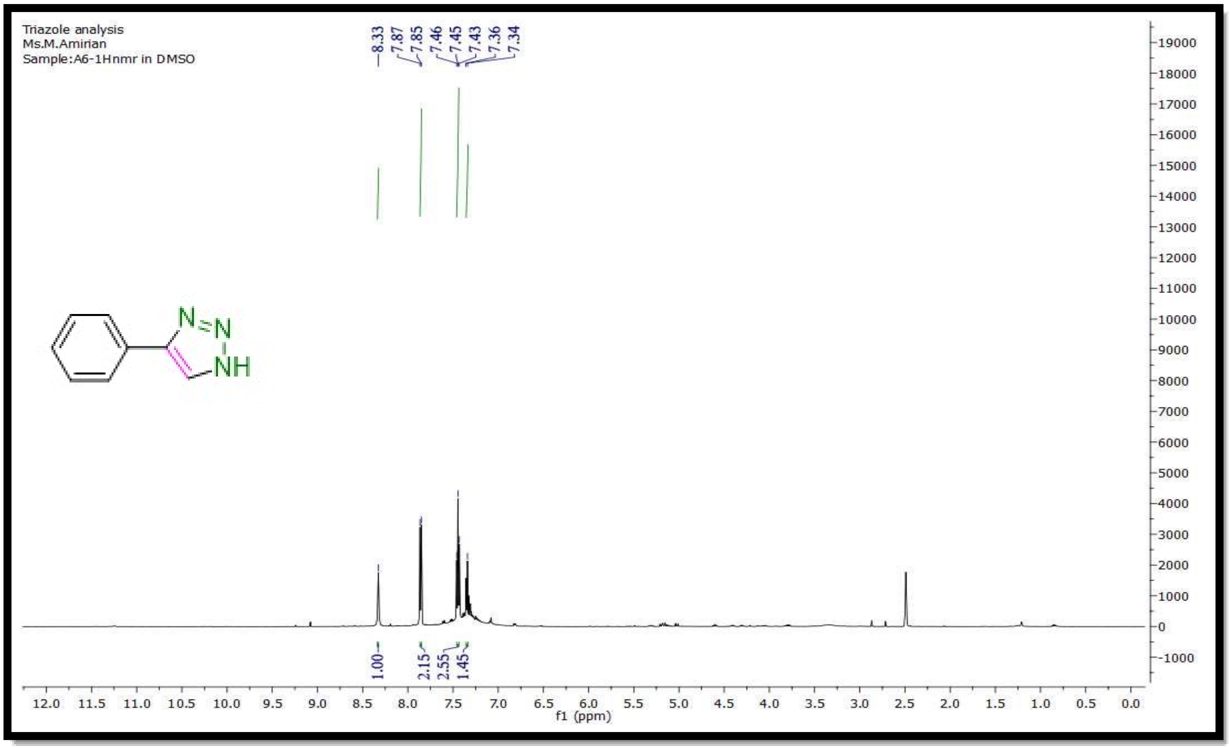

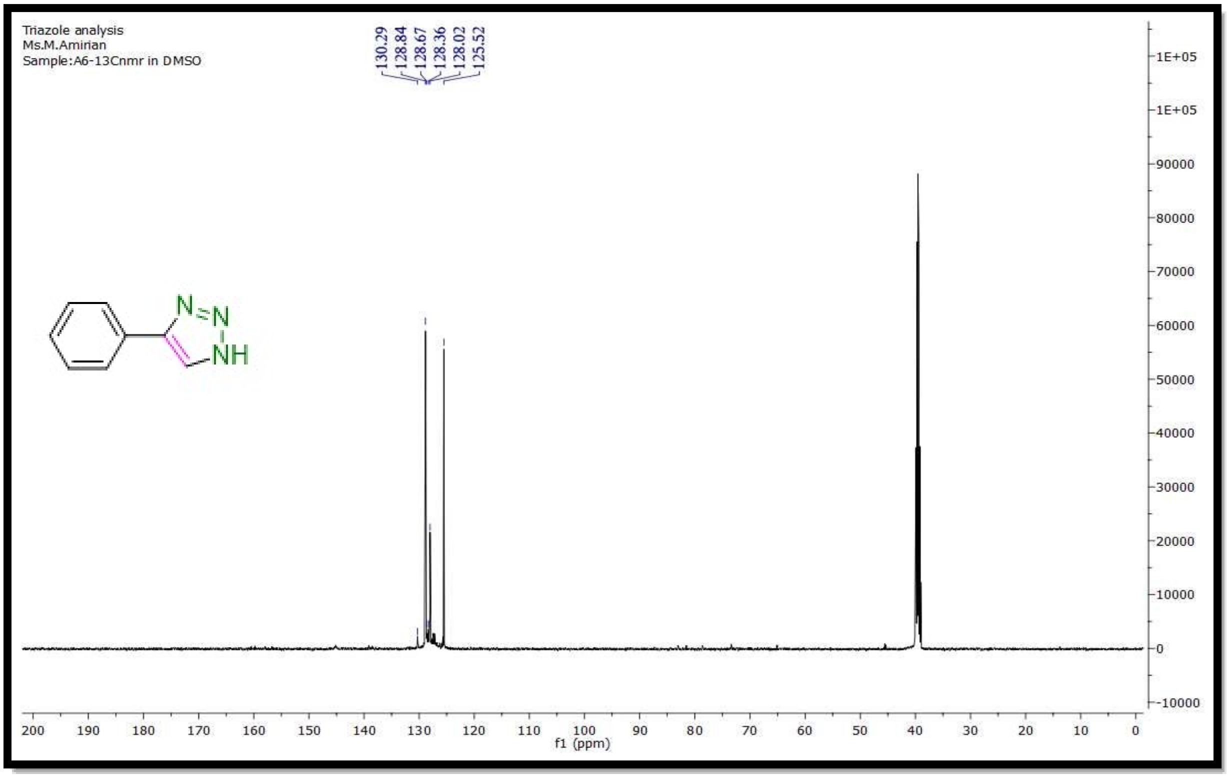

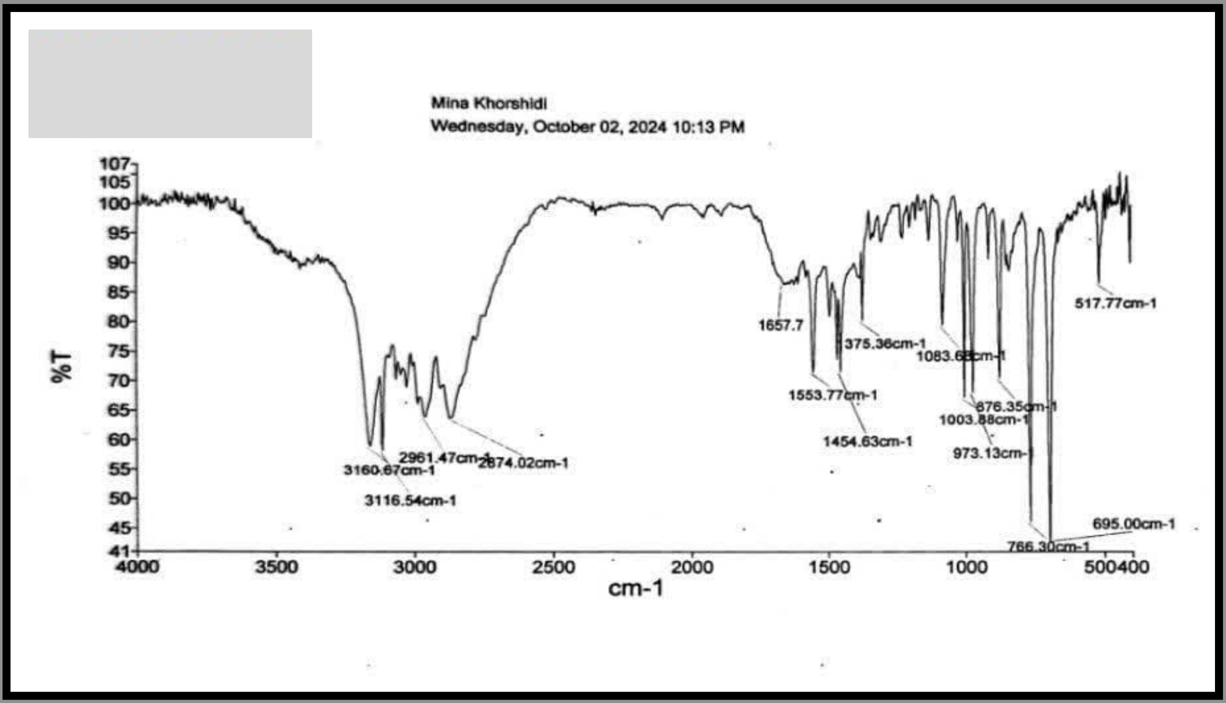
Mina Khorshidi
Wednesday, October 02, 2024 10:13 PM
%T
cm-1
3160.67cm-1
3116.54cm-1
2961.47cm-1
2874.02cm-1
1657.7
1553.77cm-1
1454.63cm-1
375.36cm-1
1083.68cm-1
1003.88cm-1
876.35cm-1
973.13cm-1
766.30cm-1
695.00cm-1
517.77cm-1

4-(2-Methoxyphenyl)-1H-1,2,3-triazole:

Melting point: 79-80 °C

FT-IR (KBr, ν, cm−1): 3395(N-H Stretch), 3002,2938(C-H), 1492,1601(C=C), 1181(C-O), 1024(C-N),

^{1}H NMR (500 MHz, DMSO) δ 8.18 (s, 1H), 7.33-7.35 (t, *J*= 5.2 Hz, 1H), 7.12-7.14 (d, *J* = 8.3 Hz, 2H), 7.02-7.05 (t, 1H), 3.90 (s, 3H).

^{13}C NMR (126 MHz, DMSO) δ 155.80, 129.21, 127.86, 127.69, 127.35, 120.70, 118.51, 111.68, 55.43.

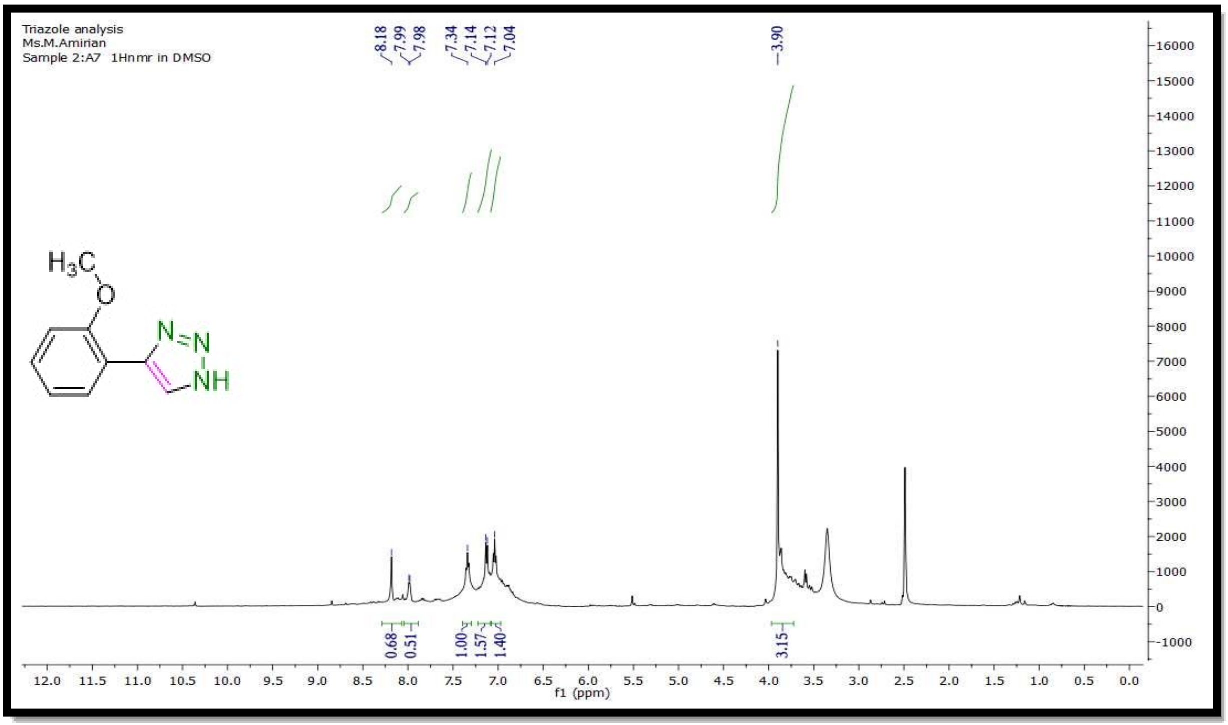

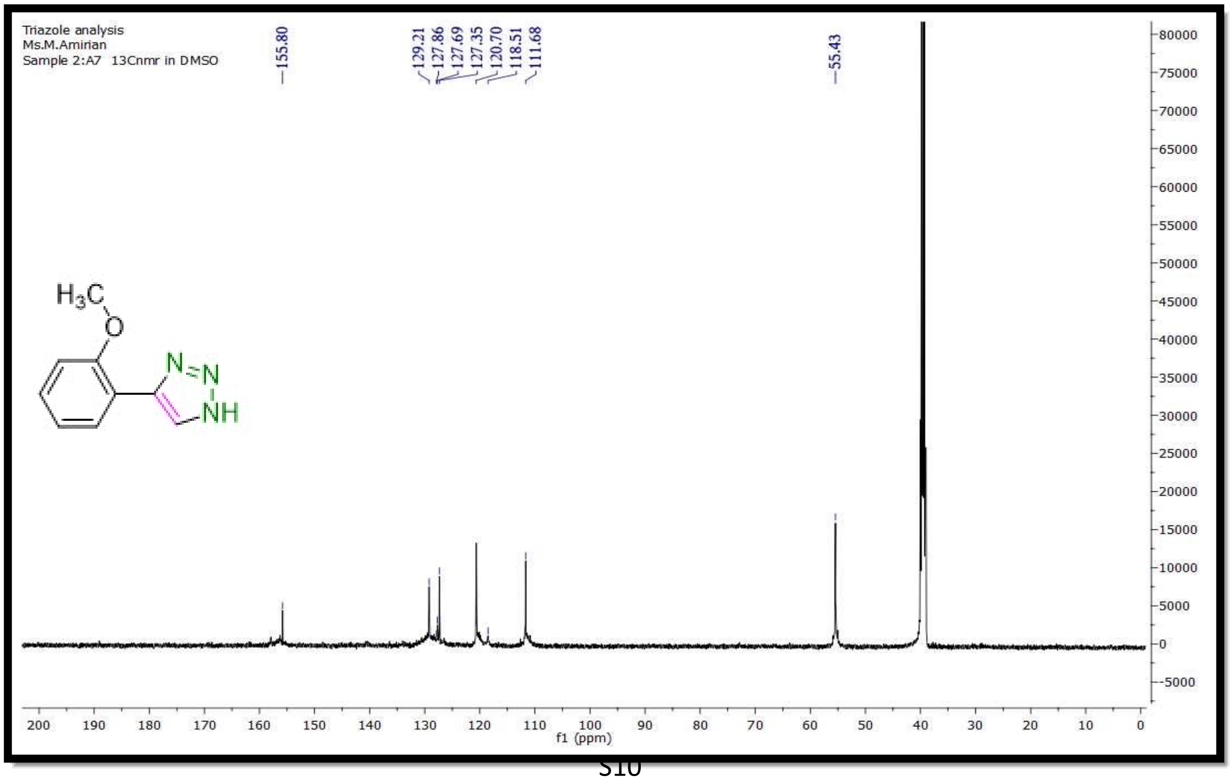

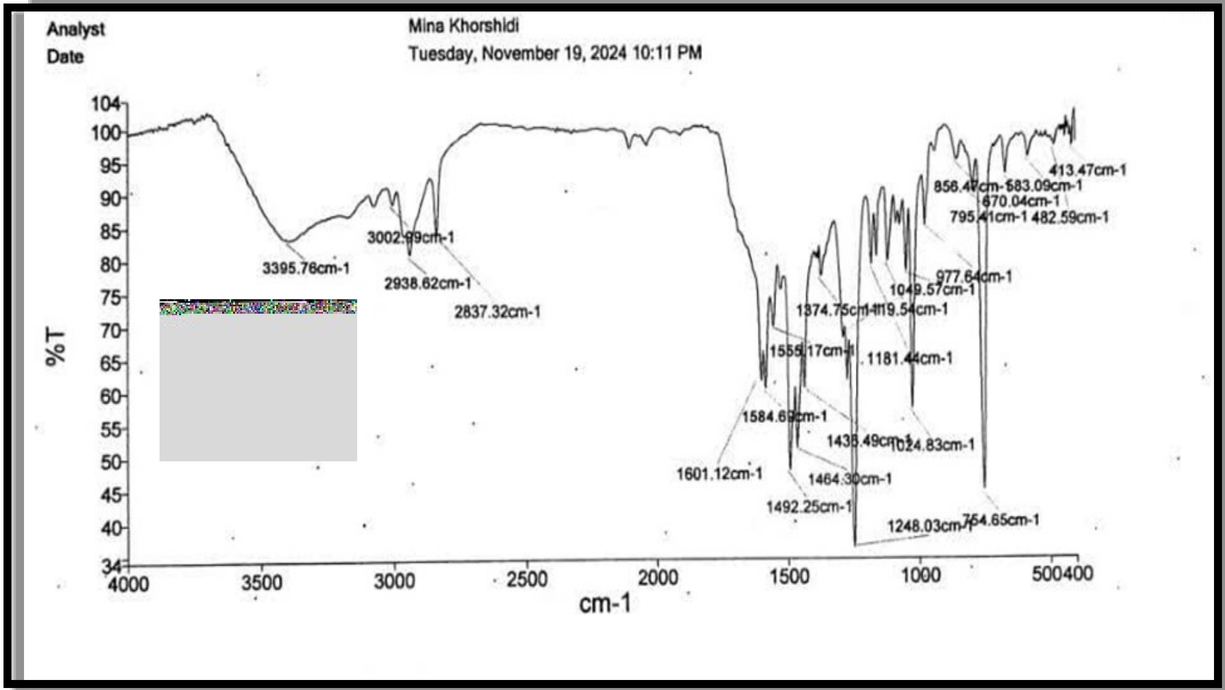
Analyst
Date
Mina Khorshidi
Tuesday, November 19, 2024 10:11 PM
%T
cm-1
3395.76cm-1
3002.99cm-1
2938.62cm-1
2837.32cm-1
1601.12cm-1
1584.69cm-1
1555.17cm-1
1492.25cm-1
1464.30cm-1
1435.49cm-1
1374.75cm-1
1119.54cm-1
1181.44cm-1
1248.03cm-1
1049.57cm-1
1024.83cm-1
977.64cm-1
856.47cm-1
795.41cm-1
754.65cm-1
683.09cm-1
670.04cm-1
482.59cm-1
413.47cm-1

4-(3-Nitrophenyl)-1H-1,2,3-triazole:

Melting point: 203-205 °C

FT-IR (KBr, ν, cm^{-1}): 3393(N-H Stretch), 3088 (C-H), 1436,1619(C=C), 1350,1528(N=O), 1099(C-N)

^{1}H NMR (500 MHz, DMSO) δ 8.65 (s, 1H), 8.61 (s, 1H), 8.30-8.31 (d, *J* = 7.8 Hz, 1H), 8.16-8.18 (d, *J* = 9.7 Hz, 1H), 7.72-7.76 (t, *J* = 8.0 Hz, 1H).

^{13}C NMR (126 MHz, DMSO) δ 148.31, 143.74, 132.27, 131.66, 130.43, 127.10, 122.42, 119.66.

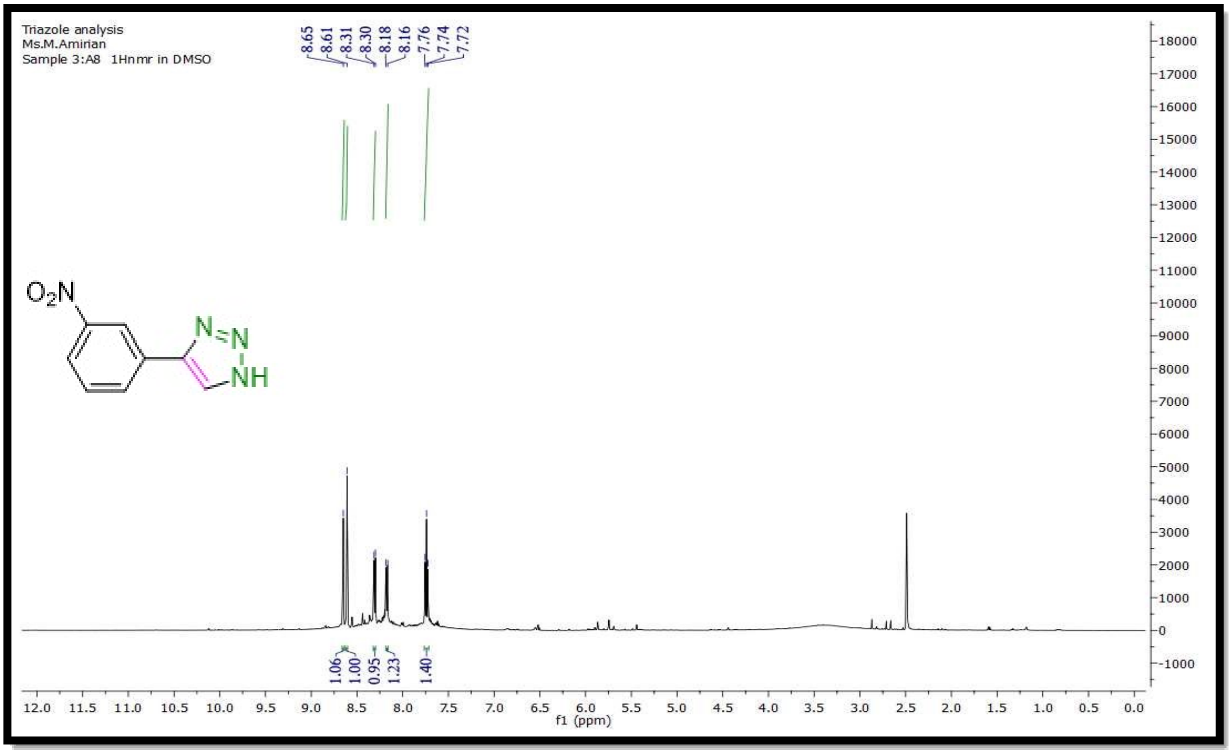

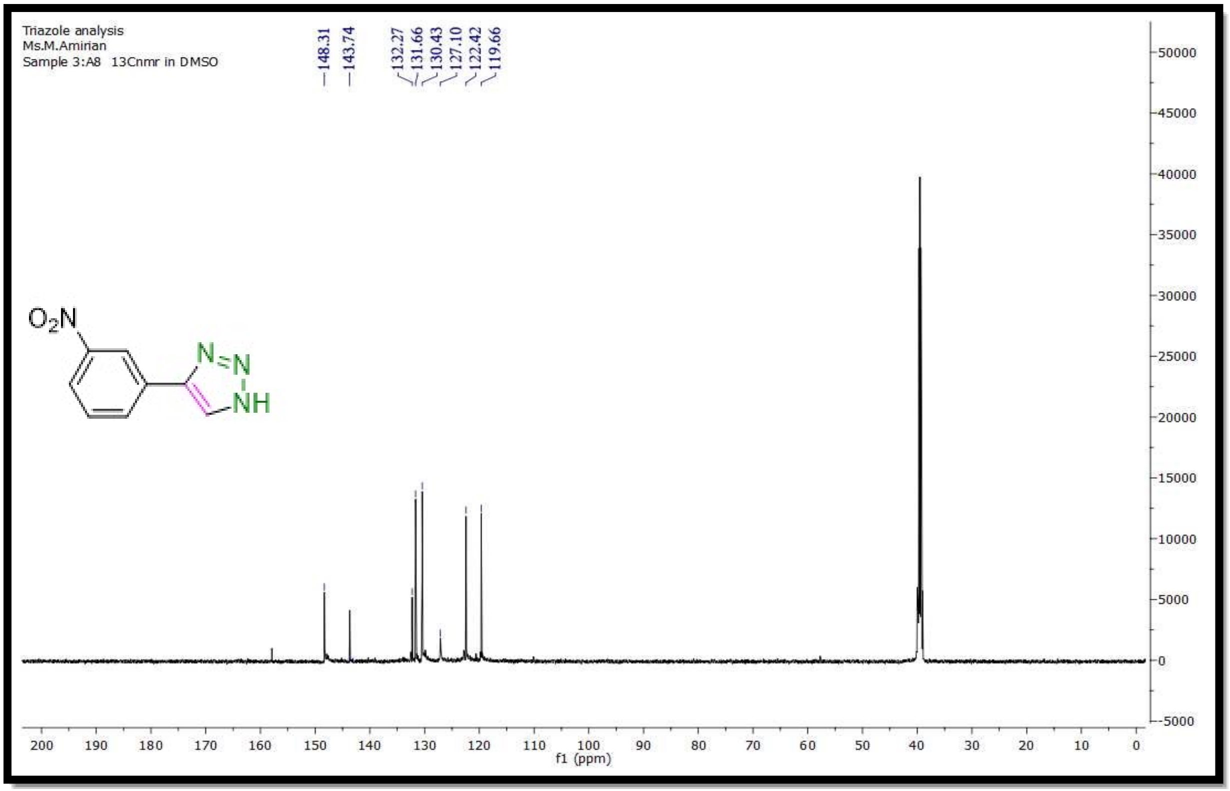

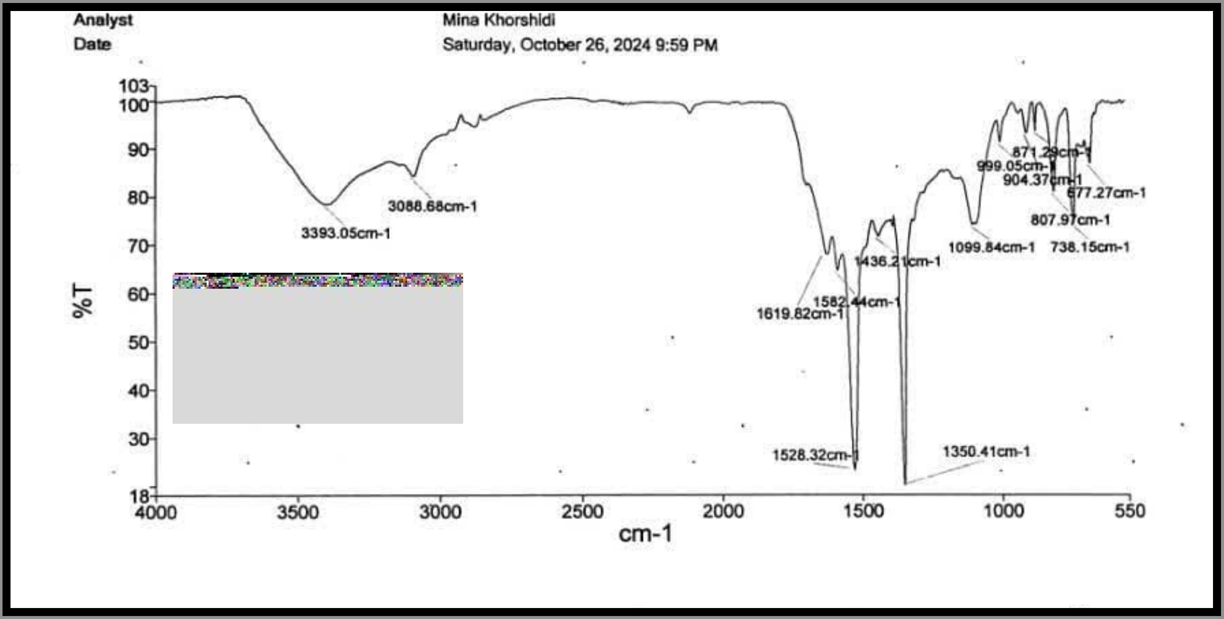
Analyst
Mina Khorshidi
Date
Saturday, October 26, 2024 9:59 PM
%T
cm-1
3393.05cm-1
3088.68cm-1
1619.82cm-1
1582.44cm-1
1528.32cm-1
1436.21cm-1
1350.41cm-1
1099.84cm-1
999.05cm-1
904.37cm-1
871.29cm-1
807.97cm-1
738.15cm-1
677.27cm-1

4-(Furan-2-yl)-1H-1,2,3-triazole:

Melting point: 64-66 °C

FT-IR (KBr, ν, cm^{-1}): 3443(N-H Stretch), 3126,2928(C-H), 1375,1622(C=C), 1502(N-H bending), 1271(C-O), 1014(C-N),

^{1}H NMR (250 MHz, DMSO-d_6) δ 8.08 (s, 1H), 7.73 (s, 2H), 6.78 (s, 1H), 6.58 (s, 1H).

^{13}C NMR (63 MHz, DMSO-d6) δ 146.37, 143.16, 138.43, 127.35, 112.07, 107.44.

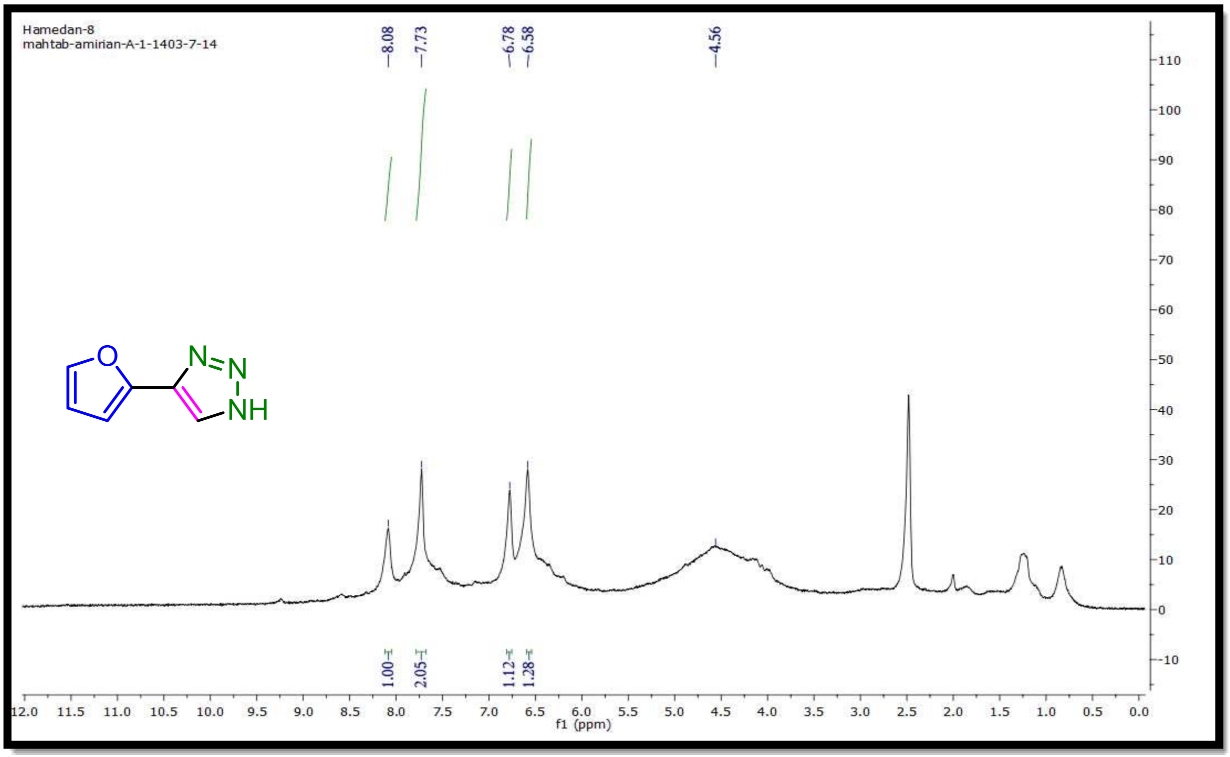

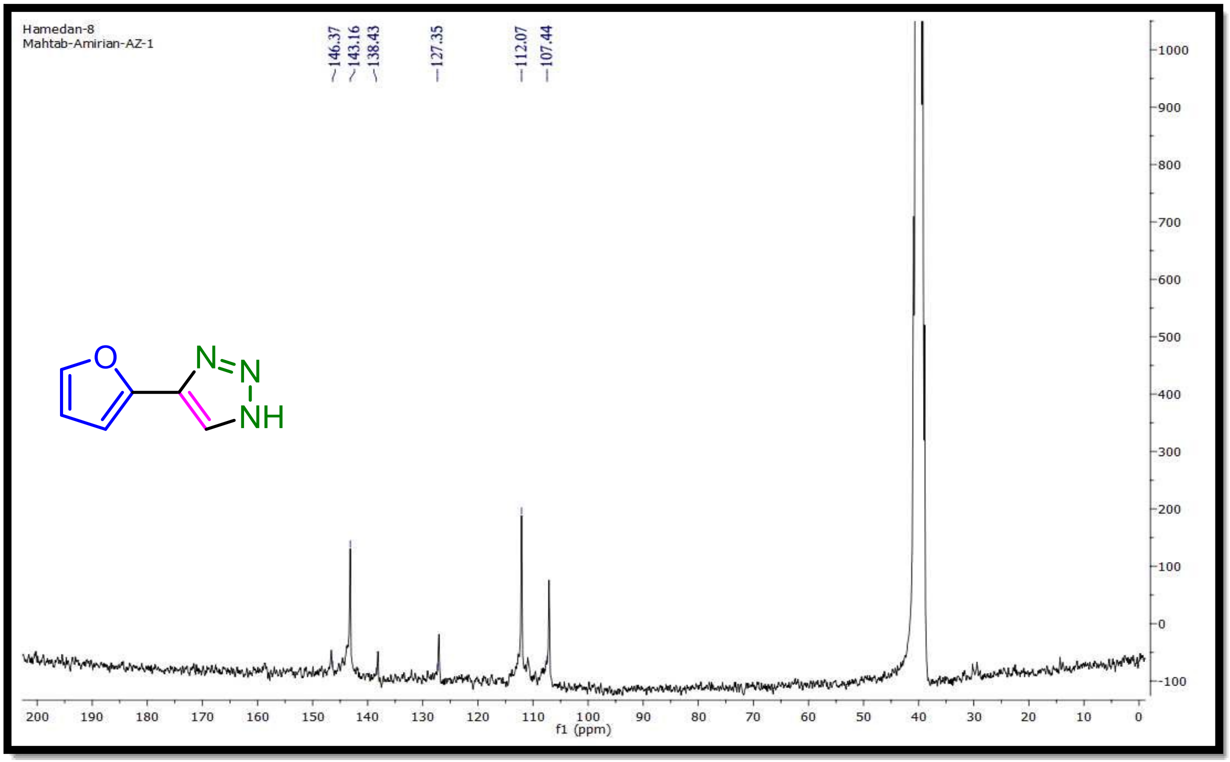

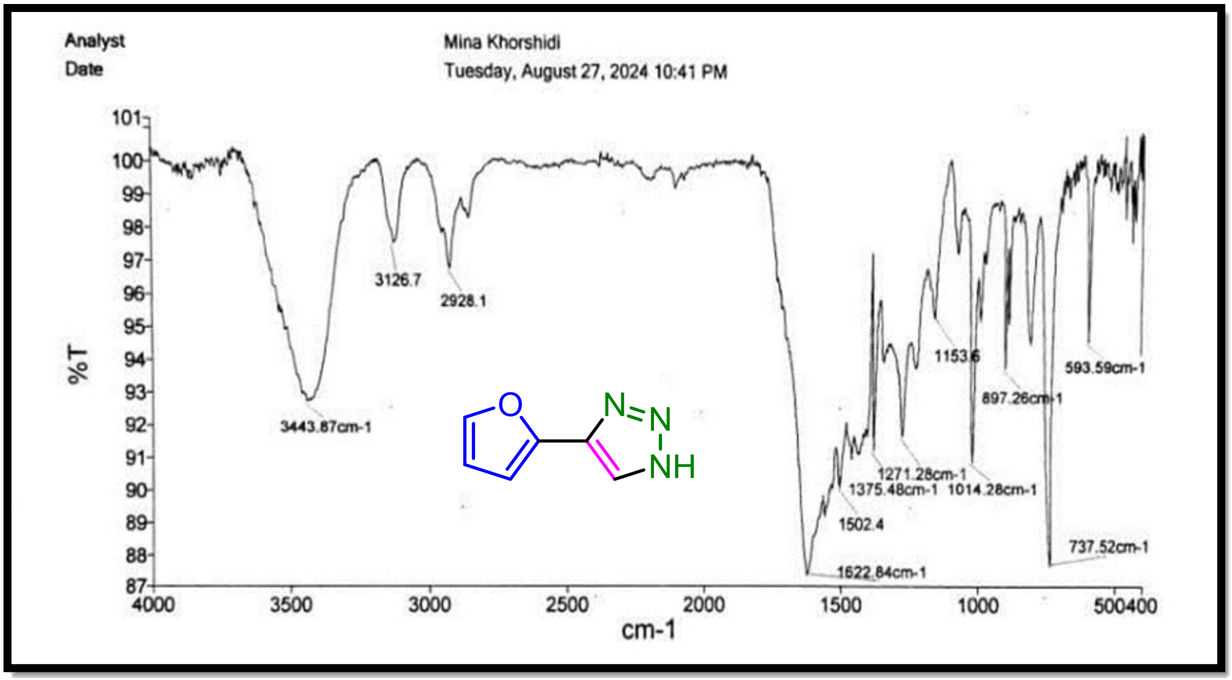
Analyst
Date
Mina Khorshidi
Tuesday, August 27, 2024 10:41 PM
%T
cm-1
3443.87cm-1
3126.7
2928.1
1622.84cm-1
1502.4
1375.48cm-1
1271.28cm-1
1153.6
1014.28cm-1
897.26cm-1
737.52cm-1
593.59cm-1
N
N
NH
O

4-(4-Methoxyphenyl)-1H-1,2,3-triazole:

Melting point: 163-164 ^{o}C

FT-IR (KBr, ν, cm^{-1}): 3422(N-H Stretch), 3163,2959(C-H), 1489,1614(C=C), 1512(N-H bending), 1252(C-O), 1031(C-N),

^{1}H NMR (250 MHz, DMSO-d_6) δ 8.15 (s, 1H), 7.73-7.76 (s, 2H), 6.96-6.99 (s, 2H), 6.85 (s, 1H), 3.75 (s, 3H).

^{13}C NMR (63 MHz, DMSO-d_6) δ 159.49, 144.96, 131.28, 127.32, 123.44, 114.75, 55.58.

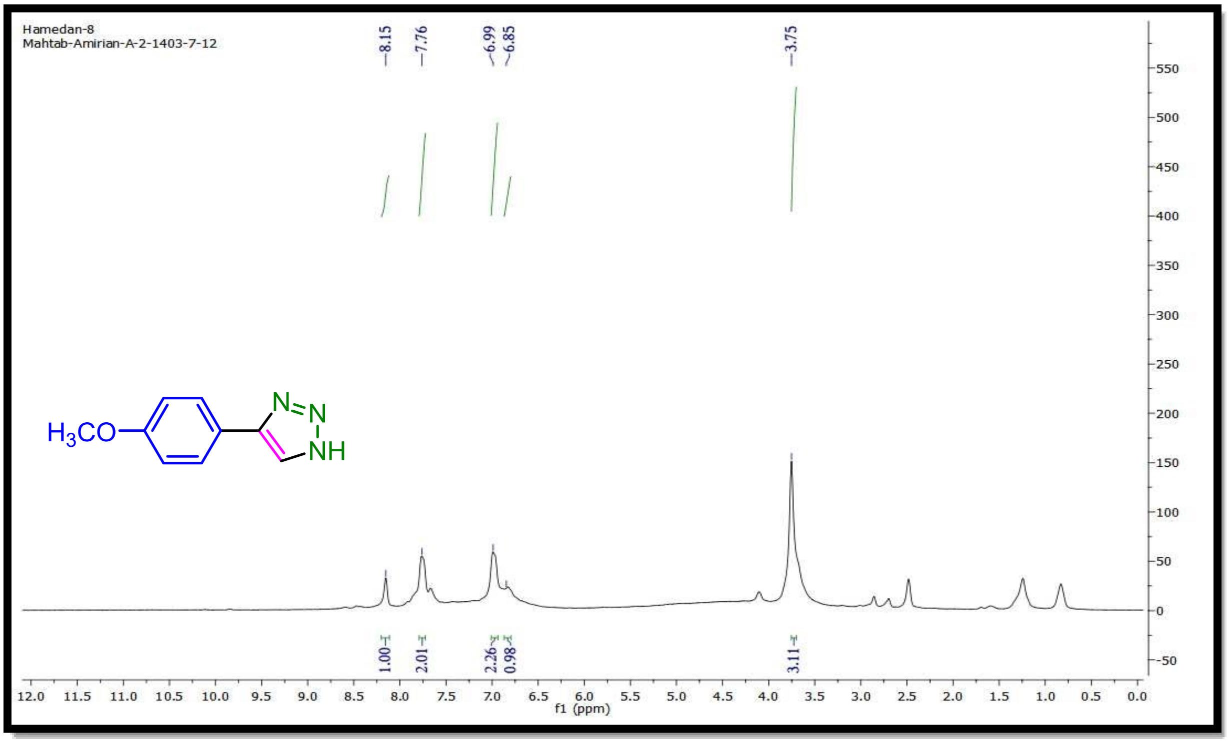

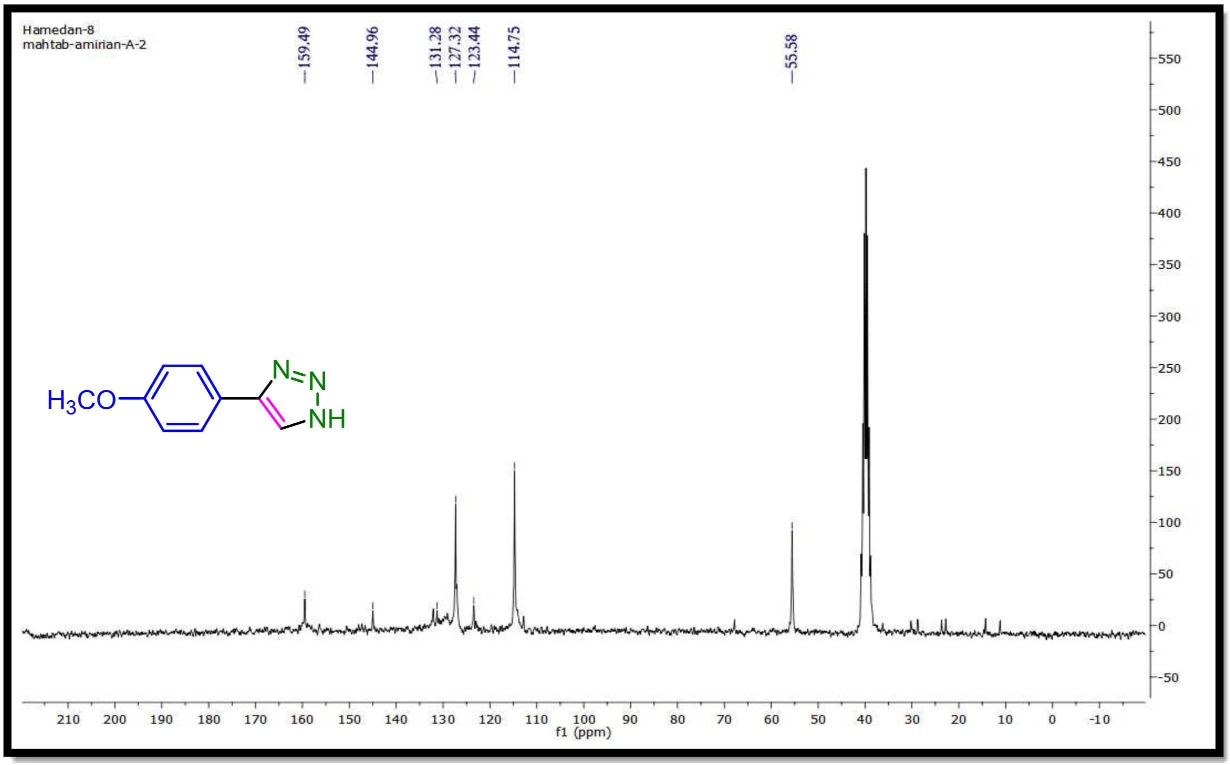

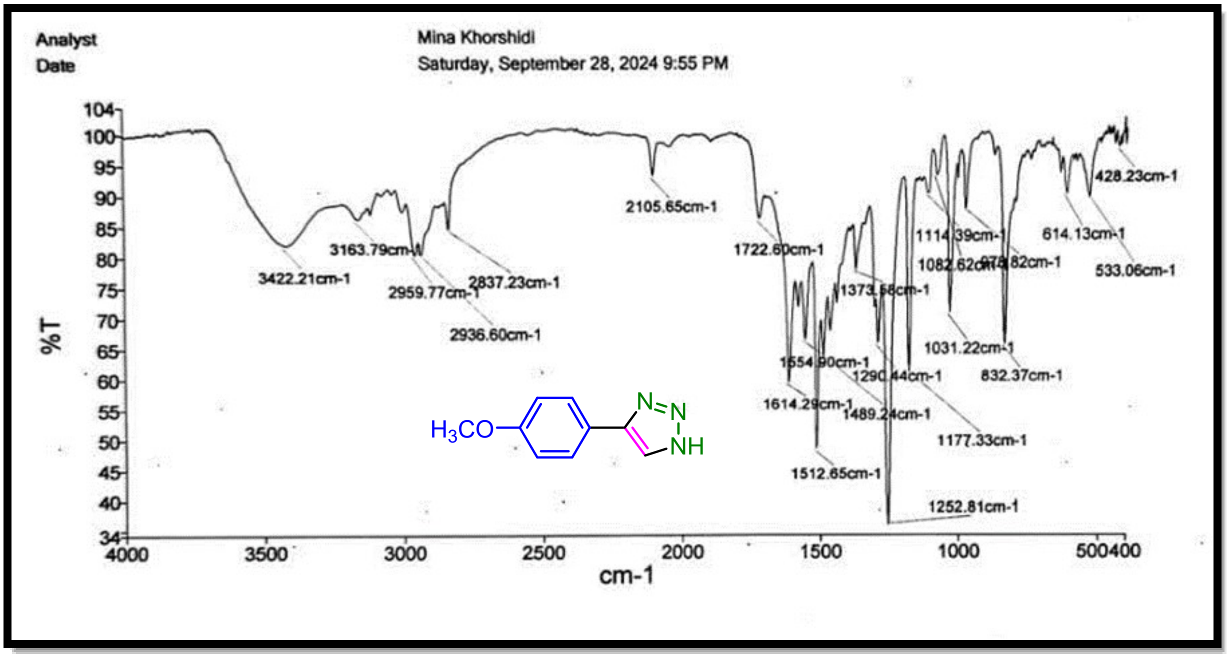

Analyst
Date
Mina Khorshidi
Saturday, September 28, 2024 9:55 PM
%T
cm-1
3422.21cm-1
3163.79cm-1
2959.77cm-1
2936.60cm-1
2837.23cm-1
2105.65cm-1
1722.60cm-1
1654.90cm-1
1614.29cm-1
1512.65cm-1
1489.24cm-1
1373.58cm-1
1290.44cm-1
1252.81cm-1
1177.33cm-1
1114.39cm-1
1082.62cm-1
1031.22cm-1
975.82cm-1
832.37cm-1
614.13cm-1
533.06cm-1
428.23cm-1
H_3CO
N
N
NH

N,N-Dimethyl-4-(1H-1,2,3-triazol-4-yl)aniline:

Melting point: 160-162 ^oC
FT-IR (KBr, ν, cm^{-1}): 3414(N-H Stretch), 2919(C-H), 1443,1614(C=C), 1522(N-H bending), 1197(C-N),
^1H NMR (500 MHz, DMSO) δ 8.09 (s, 1H), 7.64-7.66 (m, 2H), 6.76 – 6.79 (m, 2H), 2.91 (s, 6H).

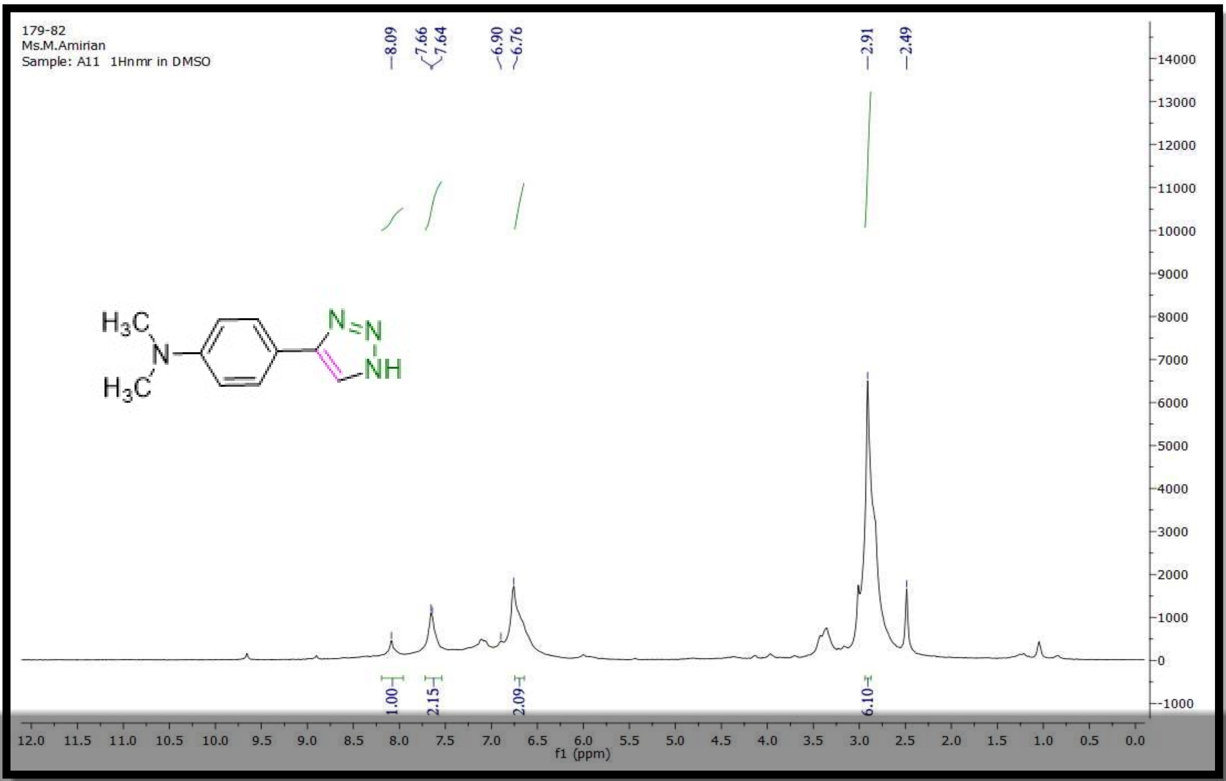

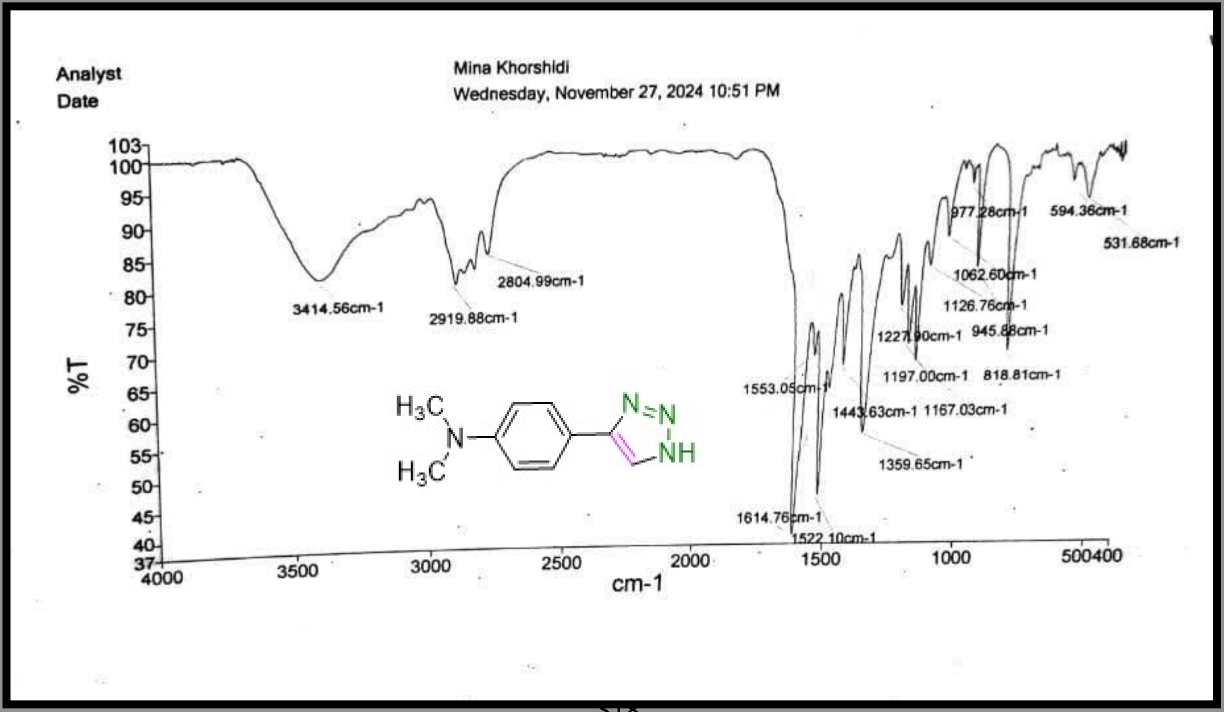

4-(4-Nitrophenyl)-1H-1,2,3-triazole:

Melting point: 198-200 ^{o}C

FT-IR (KBr, ν, cm^{-1}): 3375(N-H Stretch), 3103(C-H), 1344,1597(N=O), 1518(N-H bending), 1167(C-O), 1109(C-N),

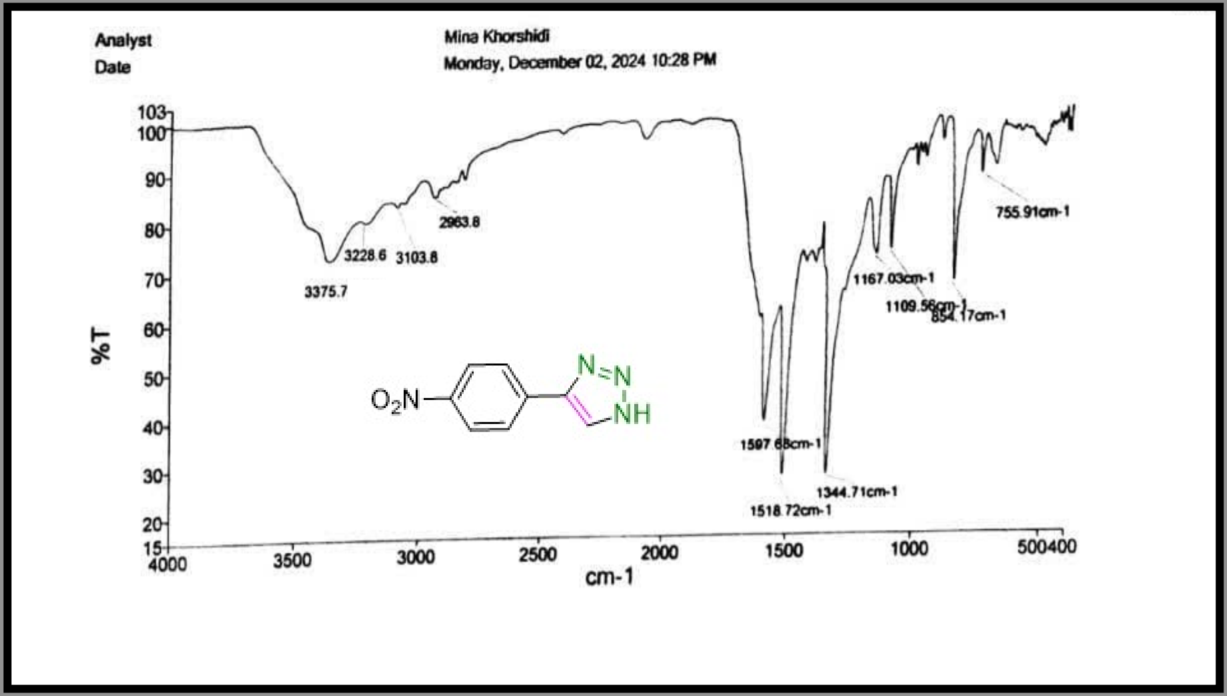

Supplement: NA-007-D5NA00299K-s001 [file NA-007-D5NA00299K-s001.pdf]
